# An Ultrasensitive Fluorescence Assay for the Detection of Halides and Enzymatic Dehalogenation

**DOI:** 10.1002/cctc.201901891

**Published:** 2020-01-31

**Authors:** Aşkın S. Aslan‐Üzel, Andy Beier, David Kovář, Clemens Cziegler, Santosh K. Padhi, Eva D. Schuiten, Mark Dörr, Dominique Böttcher, Frank Hollmann, Florian Rudroff, Marko D. Mihovilovic, Tomáš Buryška, Jiří Damborský, Zbyněk Prokop, Christoffel P. S. Badenhorst, Uwe T. Bornscheuer

**Affiliations:** ^1^ Department of Biotechnology & Enzyme Catalysis Institute of Biochemistry Greifswald University Greifswald 17487 Germany; ^2^ Loschmidt Laboratories Department of Experimental Biology and RECETOX Faculty of Science Masaryk University Brno 625 00 Czech Republic; ^3^ International Clinical Research Center St. Anne's University Hospital Brno Brno 656 91 Czech Republic; ^4^ Institute of Applied Synthetic Chemistry TU Wien Vienna 1060 Austria; ^5^ Biocatalysis and Enzyme Engineering Laboratory Department of Biochemistry School of Life Sciences University of Hyderabad Gachibowli 500046 India; ^6^ Department of Biotechnology Delft University of Technology Delft 2629 HZ (The Netherlands

**Keywords:** dehalogenase, fluorescence, halides, haloalkane, haloperoxidase

## Abstract

Halide assays are important for the study of enzymatic dehalogenation, a topic of great industrial and scientific importance. Here we describe the development of a very sensitive halide assay that can detect less than a picomole of bromide ions, making it very useful for quantifying enzymatic dehalogenation products. Halides are oxidised under mild conditions using the vanadium‐dependent chloroperoxidase from *Curvularia inaequalis*, forming hypohalous acids that are detected using aminophenyl fluorescein. The assay is up to three orders of magnitude more sensitive than currently available alternatives, with detection limits of 20 nM for bromide and 1 μM for chloride and iodide. We demonstrate that the assay can be used to determine specific activities of dehalogenases and validate this by comparison to a well‐established GC‐MS method. This new assay will facilitate the identification and characterisation of novel dehalogenases and may also be of interest to those studying other halide‐producing enzymes.

## Graphical abstract

**How sensitive**! A simple, safe, and very sensitive assay for enzymatic dehalogenation is presented. The halides produced by dehalogenases are generally very unreactive and thus hard to detect. We use hydrogen peroxide and a haloperoxidase to activate halides to hypohalous acids, which are easily detected using the sensitive fluorogenic probe aminophenyl fluorescein.

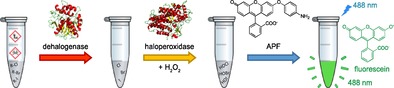

## Introduction

Chemical industries annually produce millions of tons of short‐chain haloalkanes that are used as solvents and synthetic intermediates.^1^ Additionally, each year several million tons of methyl halides, predominantly chloromethane, are released into the atmosphere by terrestrial and marine biomass.^2^ Haloalkanes are toxic and are often employed as pesticides, herbicides, and chemical warfare agents. Evaporation, improper disposal, spillage, and deliberate release contribute to contamination of the atmosphere, soil, and water with these persistent pollutants.^2^, ^3^ Biocatalytic degradation of haloalkanes by enzymes known as dehalogenases^4^ is the most promising strategy for environmental remediation.^1^, ^5^ Professor Dick B. Janssen, to whom this special issue is dedicated, discovered the enzymatic dehalogenation of haloalkanes in 1984.1a Janssen and co‐workers reported the hydrolytic dehalogenation of 1,2‐dichloroethane by crude extracts of the bacterium *Xanthobacter autotrophicus* GJ10, leading to the discovery of DhlA, the first haloalkane dehalogenase.1a, 3, 6

**Scheme 1:**
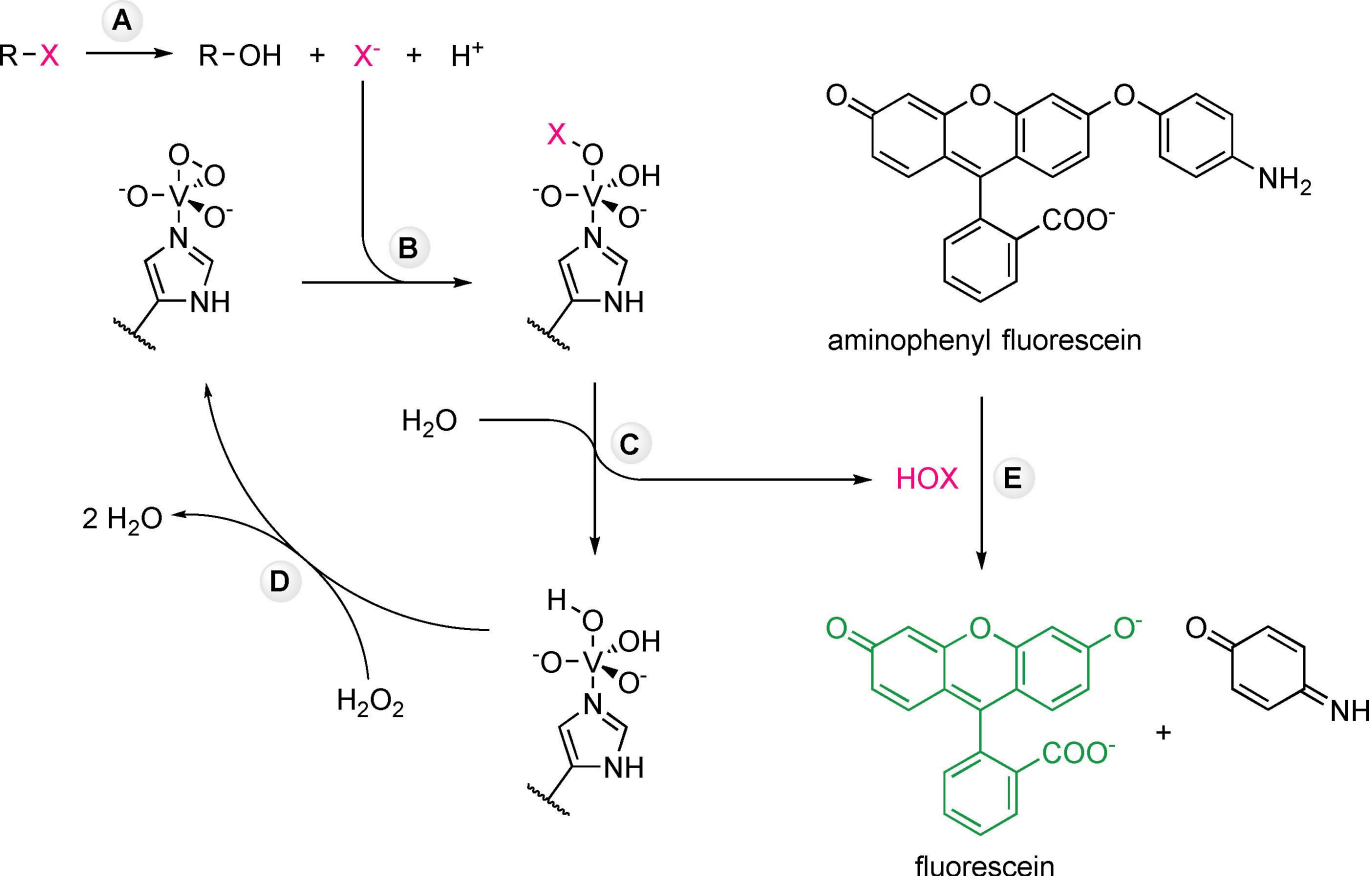
The principles behind the HOX assay for halides and dehalogenase activity. A) Haloalkane dehalogenases hydrolyse haloalkanes to the corresponding alcohols, protons and halide ions (X^−^). The halides formed are generally not very reactive but can be activated by a haloperoxidase‐catalysed two‐electron oxidation.18b, 19 In the case of vanadium‐dependent haloperoxidases the cofactor is a vanadate (V^5+^) ion coordinated by a conserved histidine residue. These enzymes are very stable because the cofactor cycles between the vanadate and peroxovanadate (oxidised) forms without changing the vanadium oxidation state. B) The halide ion and a proton react with the peroxovanadate cofactor, forming an intermediate that C) reacts with water to release the hypohalous acid (HOX). D) Hydrogen peroxide re‐oxidises the vanadate to peroxovanadate, completing the haloperoxidase catalytic cycle.18b E) Oxidation of aminophenyl fluorescein by the hypohalous acid results in the formation of fluorescein, a bright fluorescent dye that can be detected at nanomolar concentrations.

Over 40 haloalkane dehalogenases originating from diverse bacterial, archaeal, and eukaryotic species have since been described in the scientific literature.^7^ These enzymes hydrolyze a broad range of haloalkanes to the corresponding alcohols, accompanied by the release of protons and halide ions (Scheme 1).^8^ The question of how haloalkane dehalogenases evolved to degrade xenobiotic haloalkanes, both in nature and in the laboratory, has stimulated intense research for over three decades.^3^ As a result, haloalkane dehalogenases have become established as a model system for studying enzyme promiscuity, structure‐function relationships, and protein engineering by computational design or directed evolution.^9^ Of particular interest is the characterization of the thousands of putative dehalogenase sequences available in public databases7b and the identification of novel variants in large mutant and metagenome libraries.7a This creates an unmet demand for sensitive and high‐throughput assays to screen for haloalkane dehalogenase activity.^10^

Using gas chromatography and mass spectrometry (GC‐MS) to identify the alcohol products of dehalogenation reactions remains the analytical gold standard but these methods lack the throughput required for screening large libraries, especially since often several substrates need to be tested.^8^ Therefore, preliminary characterisation of haloalkane dehalogenases often relies on detection of either the protons or halide ions produced. The most popular “phenol red”^11^ and “Iwasaki”^12^ assays are based on the detection of pH changes and halide release, respectively. In 96‐well microtiter plate format the phenol red assay has a detection limit of about 500 μM protons.^11^, ^13^ More sensitive assays employing fluorescent pH indicators have recently been developed, enabling the detection of 40 to 400 μM protons.^10^ The use of very low buffer concentrations (<2 mM) means that pH is easily changed, also by factors other than enzymatic dehalogenation, reducing the reliability of pH assays. For this reason, the Iwasaki assay for halide ions has remained popular in dehalogenase assays, despite the involvement of extremely toxic mercury derivatives.7a In microtiter plate format, variations of the Iwasaki assay were reported to have detection limits for halide ions between 20 and 270 μM.10b, 14

Most of the dehalogenases described to date are not very fast enzymes, with specific activities commonly expressed in nmol/s/(mg protein).7b, 8 Hence, a significantly more sensitive assay for halide ions would likely facilitate the discovery of novel dehalogenases that could not be identified using existing assays.

Here we describe the development of a novel halide assay based on the haloperoxidase‐catalysed oxidation of chloride, bromide, and iodide (Scheme 1). We demonstrate that the new halide assay is up to three orders of magnitude more sensitive than currently available alternatives and validated it by comparison to a standard GC‐MS method for determining the specific activities of two model dehalogenases.

## Results and Discussion

### Using the vanadium‐dependent chloroperoxidase from *Curvularia inaequalis* for halide assays

One of the oldest methods for the quantification of bromide involves using the strong oxidant chloramine‐T to oxidise bromide to bromine. The bromine then reacts with fluorescein to form the intensely coloured compound eosin, which can be detected spectrophotometrically at 520 nm.^15^ Unfortunately, excess chloramine‐T can further react with the eosin, bleaching its colour and destroying the signal. This makes the assay time‐dependent and the reaction has to be stopped by the addition of sodium thiosulfate. We argue that if halides could be oxidised under milder conditions, this excessive bleaching of the dye could be avoided, resulting in a more sensitive, reliable, and quantitative assay. Enzymes are often employed for their ability to catalyse harsh reactions under mild conditions and therefore we set out to find an enzymatic process that would enable halide quantification.^16^ Halogenases and haloperoxidases are some of the few enzymes known to use halides as their natural substrates. Table S1 in the Supporting Information summarises all enzymes in the BRENDA database^17^ that use halides as substrates, including unnatural and promiscuous activities. Haloperoxidases are particularly interesting as they catalyse the two‐electron oxidation of halides, using hydrogen peroxide as a mild oxidant and forming the corresponding hypohalous acids as products (Scheme 1).^18^

Compared to halides, hypohalous acids are very reactive and can be detected easily. To develop our new halide assay, we combined the haloperoxidase‐catalysed oxidation of halides with the use of a fluorogenic probe to detect the hypohalous acids formed (Scheme 1). First, we selected a vanadium‐dependent haloperoxidase for use in the halide oxidation assay because these enzymes are very stable in the presence of hydrogen peroxide, in contrast to heme‐dependent haloperoxidases that are often inactivated by low concentrations of hydrogen peroxide.^20^

We chose the vanadium‐dependent chloroperoxidase from *Curvularia inaequalis* (*Ci*VCPO) as it is easily expressed in *Escherichia coli*, stable at room temperature, and capable of oxidising chloride, bromide, and iodide to the corresponding hypohalous acids.18a, 18d, 18e, 20, 21 We then selected aminophenyl fluorescein as the fluorogenic probe. From here on we will refer to the new assay as the “HOX assay” which is short for “halide oxidation assay” and reflects the involvement of hypohalous acids (HOX).

**Figure 1:**
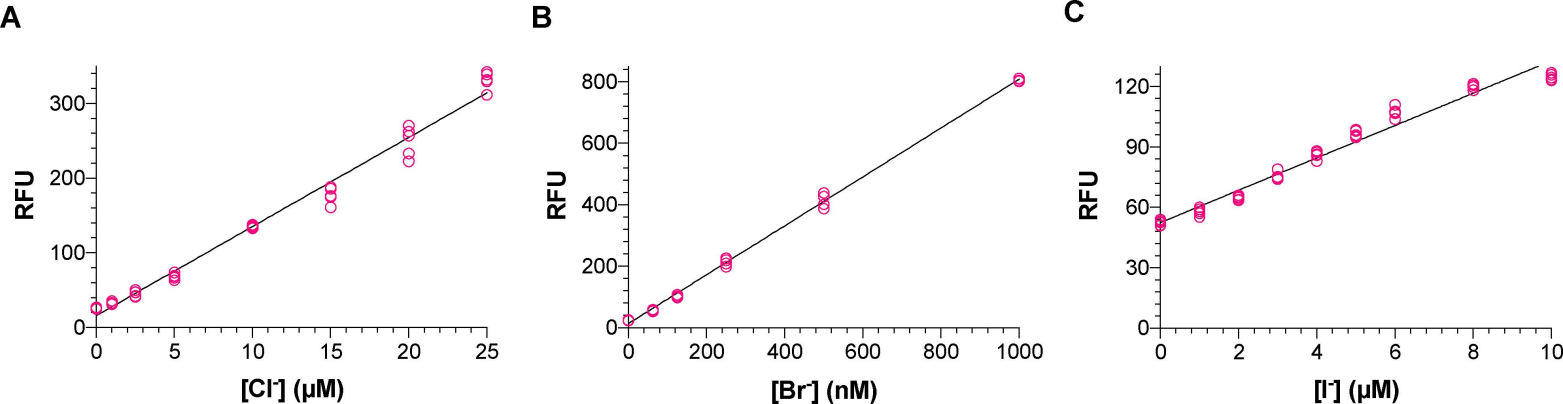
Calibration curves for A) chloride, B) bromide, and C) iodide. The HOX assay can quantify low micromolar concentrations of chloride and iodide and nanomolar concentrations of bromide. Each replicate is plotted as a separate data point (n=5) and the limits of detection are defined as the blank values plus three times the standard deviation of the blank. The detection limits, summarised in Table 1, were 1 μM for chloride, 20 nM for bromide, and 1 μM for iodide. GraphPad Prism was used for plotting data and linear regression.

We added low concentrations of chloride, bromide, or iodide to 40 μl reaction mixtures containing *Ci*VCPO, hydrogen peroxide, and aminophenyl fluorescein. As expected, fluorescence values for the controls were low and increased significantly upon addition of halides (Figure 1). The detection limit was 1 μM for both chloride (Figure 1A) and iodide (Figure 1C), allowing low‐micromolar concentrations to be quantified. A linear relationship between iodide concentration and fluorescence was observed despite lower absolute fluorescence values compared to chloride. In contrast, absolute fluorescence values for bromide were much higher than for chloride, making the assay significantly more sensitive. Nanomolar concentrations of bromide could be quantified with a detection limit of 20 nM (Figure 1B). Differences in sensitivity of the HOX assay to chloride, bromide, and iodide can be explained by different reactivities of the hypohalous acids toward aminophenyl fluorescein and HOX‐scavenging species (like protein tyrosine residues).18c, 22 In conclusion, the HOX assay allows chloride, bromide, and iodide to be detected at concentrations far below the detection limits of alternative halide assays reported so far.

### The HOX assay is much more sensitive than existing alternatives

The detection limits of the HOX assay are summarised in Table 1. The colorimetric Iwasaki assay and assays based on the collisional quenching of fluorescent dyes by halide ions are currently major alternatives to the HOX assay.^12^, ^23^ We experimentally determined the detection limits for the Iwasaki assay and a lucigenin‐based collisional quenching assay. The results are discussed below and summarised in Table 1 for comparison to the HOX assay.

**Table 1:** Summary of the halide assays used in this study and their detection limits.

	Iwasaki assay^12^	Lucigenin assay^28^	HOX assay [this work]
Principle of the assay	Halides displace thiocyanate from Hg(SCN)_2_, forming Fe(SCN)^2+^ that absorbs light at 460 nm. Absorbance at 460 nm increases with increasing halide concentration.^[12,14, 24]^	Halides reduce fluorescence of the dye lucigenin by collisional quenching. Fluorescence emission at 503 nm decreases with increasing halide concentration.^26^, ^27^, ^28^	Halides are oxidised by a haloperoxidase to hypohalous acids which are detected using aminophenyl fluorescein. Fluorescence at 525 nm increases with increasing halide concentration.
Standard curves	Figure S1	Figure S2	Figure 1
Detection limit for chloride	156 μM	49 μM	1 μM
Detection limit for bromide	29 μM	63 μM	20 nM
Detection limit for iodide	36 μM	35 μM	1 μM
Hazardous reagents	Mercuric thiocyanate is extremely toxic and is fatal when inhaled or ingested. It is very toxic to aquatic life with long‐lasting effects. It must be carefully disposed of.	Lucigenin can cause skin and eye irritation and may be harmful if inhaled, ingested, or absorbed through the skin. It is not considered very dangerous to work with.	Hydrogen peroxide must be handled with care, but no component of the assay is considered dangerous to work with.

Probably the most popular halide assay is the “Iwasaki assay” which has been adapted several times since its introduction in 1952 and has been miniaturised to the microtiter plate format.^12^, ^14^, ^24^ It is based on the displacement of thiocyanate from mercuric thiocyanate by chloride, followed by the formation of an orange‐red‐coloured ferric thiocyanate complex that can be quantified spectrophotometrically at 460 nm. The assay can also detect bromide and iodide^12^, ^14^, ^24^ and therefore the method is popular in dehalogenase assays7a despite the involvement of extremely toxic and hard‐to‐dispose‐of chemicals. We performed the Iwasaki assay in 200 μl volume in microtiter plates and obtained detection limits of 156 μM for chloride, 29 μM for bromide, and 36 μM for iodide (Table 1). Standard curves are shown in the Supporting Information (Figure S1).

The quenching of fluorescent dyes or fluorescent proteins by halide ions has been used to measure halide concentrations.^25^ Quenching of the fluorescence of 6‐methoxy‐*N*‐(3‐sulfopropyl) quinolinium (SPQ) by halides^26^ has made it a popular chloride sensor^27^ which has been employed in dehalogenase assays.^23^ SPQ responds to a broad range of concentrations up to 500 mM, but with a detection limit of 1 mM it is not useful for quantifying micromolar concentrations of halides.^23^ The dye lucigenin is more sensitive to collisional quenching by halides and therefore we determined its limits of detection for chloride, bromide, and iodide.^26^, ^27^, ^28^ We obtained detection limits of 49 μM for chloride, 63 μM for bromide, and 35 μM for iodide (Table 1). Standard curves are shown in the Supporting Information (Figure S2).

The HOX assay has a detection limit of 1 μM for chloride, making it 50 to 150‐fold more sensitive than existing halide assays. With a detection limit of 20 nM for bromide, the HOX assay is three orders of magnitude more sensitive than any existing alternative and can detect less than a picomole of bromide in a 40 μl assay, which could be further scaled down. The detection limit of 1 μM for iodide makes the HOX assay roughly 30‐fold more sensitive than existing halide assays.

### The HOX assay can be used to quantify dehalogenase activity

**Figure 2:**
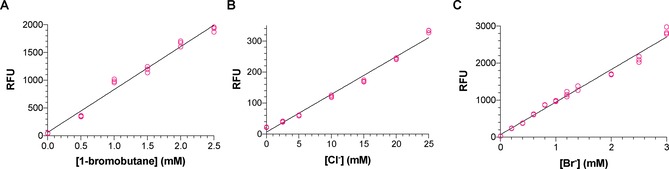
The fluorescence signal from the HOX assay is directly proportional to the amount of dehalogenase product formed. A) The dehalogenase DhaA was used to completely hydrolyse different concentrations of 1‐bromobutane. The HOX assay was then used to quantify the amount of bromide produced and a linear increase in fluorescence with increasing 1‐bromobutane concentration was observed. This demonstrated that the HOX assay is suitable for quantifying the amount of product produced by dehalogenase reactions. Standard curves for chloride (B) and bromide (C) allowed the dehalogenation of the five other substrates to be quantified as well. Each replicate is plotted as an individual data point (n=3). GraphPad Prism was used for plotting data and linear regression.

**Figure 3:**
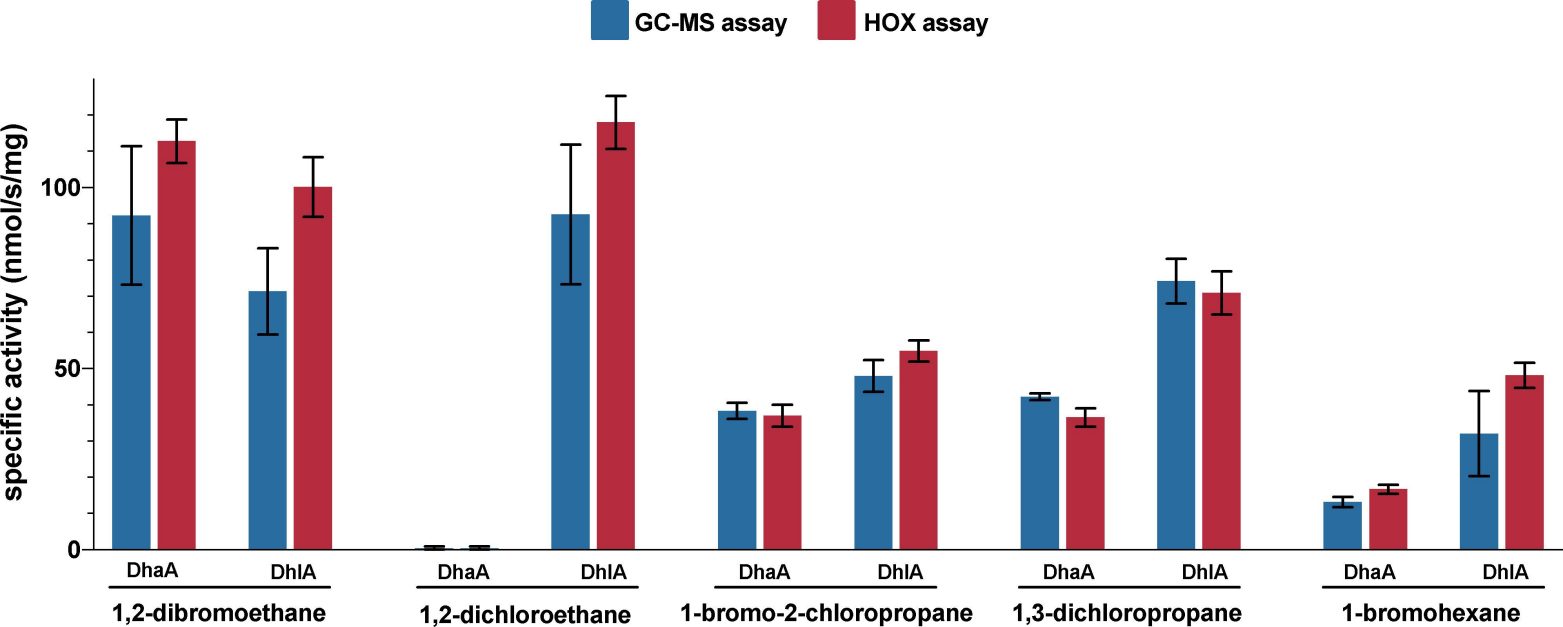
Comparison of the HOX assay to a standard GC‐MS method. The specific activities of the dehalogenases DhaA and DhlA for a number of substrates were determined using the HOX assay and a GC‐MS method. The values, expressed in nmol/s/(mg protein), reveal very good agreement between the HOX and GC‐MS assays. This result confirms that the HOX assay is reliable for the quantification of dehalogenase activity. For the HOX assay means of nine replicates are plotted with standard deviation (three reactions, each assayed in triplicate). Very small standard deviations demonstrate the excellent reproducibility of the method. For the GC‐MS assay, means of three replicates are plotted with standard deviation. GraphPad Prism was used for plotting the data.

For enzymes like dehalogenases that have inherently broad substrate specificities, the choice of substrates for detailed biochemical characterisation might not be obvious, especially since the natural substrates of the enzymes are usually not known.7a, 7b, 8, 13a Therefore, it is common to use several substrates for investigating the substrate specificity of haloalkane dehalogenases.^8^ This is especially important when comparing dehalogenase mutants, which may have subtle and unpredictable differences in substrate specificity.7a, 7b, 8, 13a Gas chromatography and mass spectrometry (GC‐MS) are often used to detect the alcohol products of dehalogenase reactions, but this is time‐consuming and therefore not suitable for analysing large numbers of variants using several substrates. As outlined above, dehalogenase assays based on the detection of protons or halides are commonly employed, but no existing assay can detect low micromolar concentrations of product formed in small‐volume reactions.^11^, 13a, 14 Our desire to detect low dehalogenase activities motivated us to develop the more sensitive HOX assay described in this paper. To demonstrate that the HOX assay is in fact suitable for detection and quantification of dehalogenase activity we used the model haloalkane dehalogenases DhlA, from *Xanthobacter autotrophicus* GJ10, and DhaA, from *Rhodococcus rhodochrous* NCIMB13064.^3^, ^6^, ^29^ The recombinant His‐tagged dehalogenases^30^ were expressed in *E. coli*, purified by immobilised metal‐affinity chromatography, and dialysed to remove chloride and imidazole from the purified proteins. The recombinant DhaA was then used to completely hydrolyse a series of 1‐bromobutane solutions (0 to 2.5 mM). These millimolar concentrations are well above the detection range of the HOX assay, since no more than 25 μM of fluorescein can be generated from the 25 μM of aminophenyl fluorescein used in the assay. However, simply diluting samples 1000‐fold into the final halide assay mixtures resulted in a linear relationship between 1‐bromobutane concentration and fluorescence (Figure 2A). It is remarkable that, despite this high dilution into a small reaction volume, the HOX assay is very reproducible, with small standard deviations over as many as 9 replicates (Figure 3). This demonstrated that the HOX assay can reliably be used to accurately quantify the amount of product formed by dehalogenase reactions (Figure 2A).

Next, we validated the HOX assay by demonstrating that it can be used to calculate specific activity values similar to those obtained using a standard GC‐MS method. We selected the typical haloalkane dehalogenase substrates 1,2‐dibromoethane, 1,2‐dichloroethane, 1,3‐dichloropropane, 1‐bromo‐3‐chloropropane, and 1‐bromohexane to determine the specific activities of DhaA and DhlA. For quantification of the chloride and bromide released from these substrates, we prepared a series of chloride and bromide concentrations in dehalogenase assay buffer (50 mM sodium phosphate, pH 8.0). These standards were treated in exactly the same way as dehalogenase reactions before determining fluorescence using the HOX assay. The resulting standard curves (Figure 2B and C) allowed us to quantify the chloride and bromide produced by dehalogenase reactions. DhaA and DhlA were then used to hydrolyse the five representative substrates and specific activities were determined using both the HOX assay and a GC‐MS assay. The values obtained using two dehalogenases and five different substrates were in excellent agreement, confirming that the HOX assay can be used to accurately quantify dehalogenase activity (Figure 3).

### The importance of buffer choice

Perhaps the most critical aspect of the HOX assay to consider is the choice of buffer. Despite initial concerns about phosphate inhibiting the *Ci*VCPO18d we found that 20 mM phosphate buffer (pH 6.0) supplemented with 1 mM of the orthovanadate cofactor was suitable for our experiments. The orthovanadate, especially in the presence of hydrogen peroxide, prevents inhibition of the enzyme by phosphate.18d Amines are known to interfere with the reaction between aminophenyl fluorescein and hypohalous acids and must therefore be excluded from reactions.18c, 31 Unfortunately, many biological buffers (Good's buffers) are amines and are thus not suitable for use with aminophenyl fluorescein in the HOX assay.^32^ For example, the 50 mM Tris‐H_2_SO_4_ used in the standard *Ci*VCPO storage buffer21a, 33 significantly inhibited the HOX assay (2.5 mM Tris‐H_2_SO_4_ in the final reactions). Desalting the *Ci*VCPO into phosphate buffer (50 mM, pH 8.0) using PD10 columns significantly increased the sensitivity of the assay. For both 1 μM bromide and 1 μM chloride, the desalting approximately doubled the fluorescence. For 5 μM bromide the same pattern was observed, while for 5 μM chloride the desalting increased the signal more than three‐fold (Figure S3). Tris‐H_2_SO_4_ must therefore be completely removed from the *Ci*VCPO preparation and our protocol now includes a dialysis step to achieve this. The observation that the effect of removing Tris‐H_2_SO_4_ was greater for chloride than for bromide can be explained by the reaction of amines with HOCl and HOBr to form chloramines and bromamines with different reactivities.

Amines can “trap” HOCl in the form of chloramines, which retain the oxidising potential of HOCl but are much less reactive toward HOX‐sensors. The more reactive bromamines, however, can often rapidly oxidise these sensors to generate a signal.^22^, ^34^ We emphasise this phenomenon because it means that there is no direct relationship between halide concentration and absolute fluorescence values and therefore a standard series, prepared in the buffer used for other samples, should always be analysed in parallel. A final comment is that the high sensitivity of the assay means that all reagents used must be as free as possible of contamination by halides. Chloride is commonly found in protein purification buffers and cell culture media, as well as in and around pH meters. Contamination with chloride is thus very easily achieved. We recommend extensively dialysing all enzymes against halide‐free buffers carefully prepared using the purest reagents and water available.

### Towards a high‐throughput, single‐cell screening assay for dehalogenase activity

The standard substrate used for initial screening of dehalogenase libraries is dibromoethane, making the detection of bromide very important.5a, 35 Initially, one of our reasons for considering the vanadium‐dependent chloroperoxidase from *Curvularia inaequalis* (*Ci*VCPO) was its high activity in the presence of 50 nM bromide.^33^ This suggested that the *Ci*VCPO could be used for the detection of nanomolar concentrations of bromide, which turned out to be true (Figure 1, Table 1). The ability to detect nanomolar concentrations of bromide will facilitate the development of ultrahigh‐throughput screening assays, since ultimately the activities of single cells may need to be detected.^36^

## Conclusions

The two distinct topics discussed in this paper, haloalkane dehalogenases and halide assays, are united by the unmet demand for more powerful dehalogenase assays.^10^ We have developed a novel halide assay based on the biocatalytic oxidation of halides under mild conditions. The HOX assay is up to three orders of magnitude more sensitive and much safer than the currently most widely used methods employing hazardous mercury compounds. We are convinced that the HOX assay will facilitate the discovery and directed evolution of novel dehalogenases, enabling a deeper understanding of the function, natural diversity, and evolutionary history of these fascinating biocatalysts. Even without the use of ultrahigh‐throughput screening, protein engineering has resulted in impressive achievements like the creation of novel trans‐halogenases and promiscuous enzymes with both dehalogenase and luciferase activity.9c, 37 It is genuinely exciting to imagine what could be achieved using a HOX‐based ultrahigh‐throughput assay for screening large dehalogenase libraries. We suspect that the HOX assay might also be of interest to those studying other enzymes like halogenases and the promiscuous activities of more than forty enzymes are listed in Table S1.

## Experimental Section

Methods are summarised in this section and detailed protocols are provided in the Supporting Information.

### Materials

All chemicals were purchased from Merck (Merck KGaA, Darmstadt, Germany) or Roth (Carl Roth GmbH+Co. KG, Karlsruhe, Germany) unless otherwise stated. Aminophenyl fluorescein was originally purchased from Merck or the Cayman Chemical Company (distributed by Biomol GmbH, Hamburg, Germany) and subsequently synthesised as described in the literature.^38^ Detailed protocols for the synthesis and purification of aminophenyl fluorescein are provided in Section S2 of the Supporting Information.

### Protein expression and purification

The vanadium‐dependent chloroperoxidase from *Curvularia inaequalis* was recombinantly expressed in *E. coli* BL21(DE3) from the pBADVCPO vector.^33^ The protein was purified as described in literature and dialysed to remove chloride and Tris‐H_2_SO_4_. Its specific activity was determined using the monochlorodimedone assay as described in the literature.18e, 39 The His‐tagged dehalogenases DhaA and DhlA were expressed in *E. coli* BL21(DE3) from the vectors pET21b‐DhaA and pET11a‐DhlA as previously described.^30^ The proteins were purified by immobilised metal‐affinity chromatography and dialysed to remove the chloride and imidazole contained in the IMAC purification buffers.

### Halide assays

HOX assay reactions were 40 μl in volume and contained 2 mM H_2_O_2_, 25 μM aminophenyl fluorescein, 1 mM orthovanadate, 2.5 U/ml *Ci*VCPO, and variable halide concentrations in 20 mM sodium phosphate (pH 6.0 for chloride and bromide, pH 6.5 for iodide). Reactions were incubated in black 384‐well microtiter plates for 60 min at room temperature before measuring fluorescence at 525 nm (excitation at 488 nm). Standard curves (Figure 1 and Figure 2) were prepared as described in Method S1.4 of the Supporting Information. Halide assays for quantification of dehalogenase activity required samples to be diluted as described in Method S1.7 in the Supporting Information. Experimental protocols for the Iwasaki assay (Method S1.5) and the lucigenin assay (Method S1.6) are described in the Supporting Information.

### Dehalogenase assays

Dehalogenase assays were performed in 1 ml of 50 mM sodium phosphate buffer (pH 8.0) containing 10 mM of either 1,2‐dibromoethane, 1,2‐dichloroethane, 1,3‐dichloropropane, 1‐bromo‐3‐chloropropane, 1‐bromobutane, or 1‐bromohexane. Reactions were initiated by the addition of 10 μg purified DhaA or DhlA and then incubated at 30 °C, shaking at 800 rpm, for 30 to 90 min. Reactions were terminated by the addition of phosphoric acid and then either diluted for the HOX assay or extracted using *tert*‐butyl methyl ether for GC‐MS analysis. Standard curves for chloride and bromide (Figure 2B and C) and the alcohol products 2‐bromoethanol, 2‐chloroethanol, 3‐chloro‐1‐propanol, and 1‐hexanol (Figures S4–S8 in the Supporting Information) were used for quantification of dehalogenase products and specific activities.

## Conflict of interest

The authors declare no conflict of interest.

## Supporting information

As a service to our authors and readers, this journal provides supporting information supplied by the authors. Such materials are peer reviewed and may be re‐organized for online delivery, but are not copy‐edited or typeset. Technical support issues arising from supporting information (other than missing files) should be addressed to the authors.

Supplementary
