# Supplementary material for: An Ultrasensitive Fluorescence Assay for the Detection of Halides and Enzymatic Dehalogenation
Source: ChemCatChem. 2020 Jan 31;12(7):2032–9. doi: 10.1002/cctc.201901891 (PMC7188320; doi:10.1002/cctc.201901891)
Supplement: Supplementary file 1 — Supplementary [file CCTC-12-2032-s001.pdf]

ChemCatChem

Supporting Information

An Ultrasensitive Fluorescence Assay for the Detection of Halides and Enzymatic Dehalogenation

Aşkın S. Aslan-Üzel, Andy Beier, David Kovář, Clemens Cziegler, Santosh K. Padhi, Eva D. Schuiten, Mark Dörr, Dominique Böttcher, Frank Hollmann, Florian Rudroff, Marko D. Mihovilovic, Tomáš Buryška, Jiří Damborský, Zbyněk Prokop, Christoffel P. S. Badenhorst,* and Uwe T. Bornscheuer*© 2019 The Authors. Published by Wiley-VCH Verlag GmbH & Co. KGaA.
This is an open access article under the terms of the Creative Commons Attribution License, which permits use, distribution and reproduction in any medium, provided the original work is properly cited.This publication is part of a joint Special Collection with ChemBioChem on "". Please follow the link for more articles in the collection.

Supporting Information

An ultrasensitive fluorescence assay for detection of halides and enzymatic dehalogenation

Aşkın S. Aslan-Üzel,[a] Andy Beier,[b, c] David Kovář,[b, c] Clemens Cziegler,[d] Santosh K. Padhi,[e] Eva D. Schuiten,[a] Mark Dörr,[a] Dominique Böttcher,[a] Frank Hollmann,[f] Florian Rudroff,[d] Marko D. Mihovilovic,[d] Tomáš Buryška,[b] Jiří Damborský,[b, c] Zbyněk Prokop,[b, c] Christoffel P. S. Badenhorst,*[a] and Uwe T. Bornscheuer*[a]

Author Affiliations

[a] Department of Biotechnology & Enzyme Catalysis, Institute of Biochemistry, Greifswald University, Felix-Hausdorff-Str. 4, 17487 Greifswald, Germany

* uwe.bornscheuer@uni-greifswald.de; chris.badenhorst@uni-greifswald.de

[b] Loschmidt Laboratories, Department of Experimental Biology and RECETOX, Faculty of Science, Masaryk University, Kamenice 5, Bld. A13, 625 00 Brno, Czech Republic.

[c] International Clinical Research Center, St. Anne's University Hospital Brno, Pekarska 53, 656 91 Brno, Czech Republic

[d] Institute of Applied Synthetic Chemistry, TU Wien, Getreidemarkt 9, 163-OC, 1060 Vienna, Austria

[e] Biocatalysis and Enzyme Engineering Laboratory, Department of Biochemistry, School of Life Sciences, University of Hyderabad, Prof. C. R. Rao Road, Gachibowli, Hyderabad, 500046, India

[f] Department of Biotechnology, Delft University of Technology, van der Maasweg 9, 2629 HZ, Delft, The Netherlands

Table of Contents

**Table S1**	A list of enzymes that use or produce halides according to the BRENDA database	S4
**Figure S1**	Standard curves for the Iwasaki assay	S5
**Figure S2**	Standard curves for the lucigenin assay	S5
**Figure S3**	Inhibition of the HOX assay by Tris-H_2SO_4	S5
**Section S1**	**Detailed Methods**	S6
**Method S1.1**	Expression and purification of *Ci*VCPO	S6
**Method S1.2**	Determining the specific activity of *Ci*VCPO	S6
**Method S1.3**	Expression and purification of recombinant DhaA and DhlA	S7
**Method S1.4**	Fluorescence (HOX) assay using *Ci*VCPO and aminophenyl fluorescein	S7
**Method S1.5**	Iwasaki assay	S7
**Method S1.6**	Lucigenin assay	S8
**Method S1.7**	Dehalogenase assays	S8
**Method S1.8**	GC-MS analysis of dehalogenase reaction products	S9
**Figure S4**	GC-MS analysis of a 2-bromoethanol product standard	S10
**Figure S5**	GC-MS analysis of a 2-chloroethanol product standard	S10
**Figure S6**	GC-MS analysis of a 3-chloro-1-propanol product standard	S11
**Figure S7**	GC-MS analysis of a 1-hexanol product standard	S11
**Figure S8**	Standard curves for 2-bromoethanol, 2-chloroethanol, 3-chloro-1-propanol, and 1-hexanol	S12
**Section S1.9**	Protein sequences	S13
**Section S2**	**Synthesis of aminophenyl fluorescein**	S14
**Scheme S1**	Synthesis of aminophenyl fluorescein from fluorescein	S14
**Method S2.1**	NMR spectroscopy	S14
**Method S2.2**	Chromatographic methods	S15
**Method S2.3**	Melting point determination	S15
**Method S2.4**	Synthesis of 3'-hydroxy-6'-(4-nitrophenoxy)-3H-spiro[isobenzofuran-1,9'-xanthen]-3-one	S15
**Method S2.5**	Synthesis of 3'-(4-aminophenoxy)-6'-hydroxy-3H-spiro[isobenzofuran-1,9'-xanthen]-3-one	S16
**Section S2.1**	NMR spectra	S17

**Figure S9**	^{1}H-NMR of compound **[2]**	S17
**Figure S10**	^{1}H-NMR of compound **[2]** enlarged between 6.4 ppm and 8.5 ppm	S18
**Figure S11**	^{13}C-NMR of compound **[2]**	S19
**Figure S12**	^{13}C-NMR of compound **[2]** enlarged between 80 ppm and 175 ppm	S20
**Figure S13**	^{1}H-NMR of compound **[3]**	S21
**Figure S14**	^{1}H-NMR of compound **[3]** enlarged between 6.0 ppm and 8.5 ppm	S22
**Figure S15**	^{13}C-NMR of compound **[3]**	S23
**Figure S16**	^{13}C-NMR of compound **[3]** enlarged between 80 ppm and 180 ppm	S24
**Section S2.2**	List of abbreviations and chemical formulas	S25
**References**		S26

Supplementary Table S1: A list of enzymes that use or produce halides according to the BRENDA[a] database

		Substrates			Products		
**E.C. #**	**Enzyme Name**	**Br^-**	**Cl^-**	**I^-**	**Br^-**	**Cl^-**	**I^-**
1.10.3.2	Laccase			x			
1.11.1.10	Chloride peroxidase		x				
1.11.1.11	L-ascorbate peroxidase			x			
1.11.1.7	Peroxidase			x			
1.11.1.8	Iodide peroxidase			x			
1.11.1.B2	Chloride peroxidase (vanadium-containing)	x	x				
1.11.2.1	Unspecific peroxygenase	x					
1.11.2.2	Myeloperoxidase		x				
1.13.11.49	Chlorite O_2-lyase		x			x	
1.13.11.64	5-nitrosalicylate dioxygenase					x	
1.14.12.13	2-halobenzoate 1,2-dioxygenase				x	x	x
1.14.13.1	Salicylate 1-monooxygenase						x
1.14.13.22	Cyclohexanone monooxygenase			x			
1.14.13.50	Pentachlorophenol monooxygenase					x	x
1.14.19.49	Tetracycline 7-halogenase		x				
1.14.19.55	4-hydroxybenzoate brominase (decarboxylating)	x					
1.14.19.56	1H-pyrrole-2-carbonyl-[peptidyl-carrier protein] chlorinase		x				
1.14.19.57	1H-pyrrole-2-carbonyl-[peptidyl-carrier protein] brominase	x					
1.14.19.58	Tryptophan 5-halogenase		x				
1.14.19.59	Tryptophan 6-halogenase	x	x				
1.14.19.9	Tryptophan 7-halogenase	x	x				
1.14.20.14	Hapalindole-type alkaloid chlorinase	x	x				
1.21.1.1	Iodotyrosine deiodinase	x	x	x			x
1.21.1.2	2,4-dichlorobenzoyl-CoA reductase					x	
1.21.99.3	Thyroxine 5-deiodinase			x			x
1.21.99.4	Thyroxine 5'-deiodinase			x			x
1.21.99.5	Tetrachloroethene reductive dehalogenase		x			x	x
2.1.1.165	Methyl-halide transferase	x	x	x			
2.1.1.9	Thiol *S*-methyltransferase	x	x	x			
2.5.1.18	Glutathione transferase				x	x	x
2.5.1.47	Cysteine synthase					x	
2.5.1.63	Adenosyl-fluoride synthase		x				
2.5.1.94	Adenosyl-chloride synthase	x	x	x		x	
3.1.1.42	Chlorogenate hydrolase				x		
3.8.1.10	2-haloacid dehalogenase (configuration-inverting)				x	x	
3.8.1.2	(*S*)-2-haloacid dehalogenase				x	x	x
3.8.1.3	Haloacetate dehalogenase					x	
3.8.1.5	Haloalkane dehalogenase				x	x	x
3.8.1.6	4-chlorobenzoate dehalogenase					x	
3.8.1.7	4-chlorobenzoyl-CoA dehalogenase				x	x	x
3.8.1.8	Atrazine chlorohydrolase					x	
3.8.1.9	(*R*)-2-haloacid dehalogenase				x	x	
4.4.1.16	Selenocysteine lyase					x	
4.5.1.1	DDT-dehydrochlorinase					x	
4.5.1.2	3-chloro-D-alanine dehydrochlorinase					x	
4.5.1.3	Dichloromethane dehalogenase					x	
4.5.1.4	L-2-amino-4-chloropent-4-enoate dehydrochlorinase					x	

[a] BRENDA[1] is available at www.brenda-enzymes.org

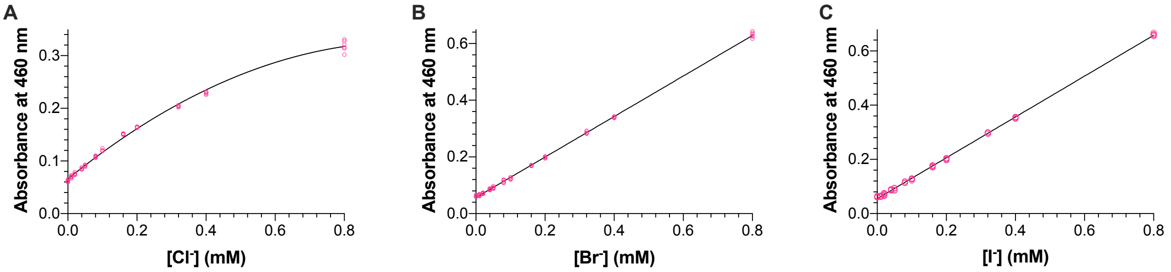

Figure S1: Standard curves for the Iwasaki assay. Absorbance at 460 nm is plotted against concentrations of **A)** chloride, **B)** bromide, and **C)** iodide. For chloride the relationship between absorbance at 460 nm and concentration is quadratic. For bromide and iodide, the relationship is linear. Each replicate is plotted as an individual data point (n=6). Detection limits are defined as the blank value plus three times the standard deviation of the blank. These values are 156 µM for chloride, 29 µM for bromide, and 36 µM for iodide. Detection limits are summarised in Table 1. GraphPad Prism was used for plotting and fitting data to linear or quadratic models.

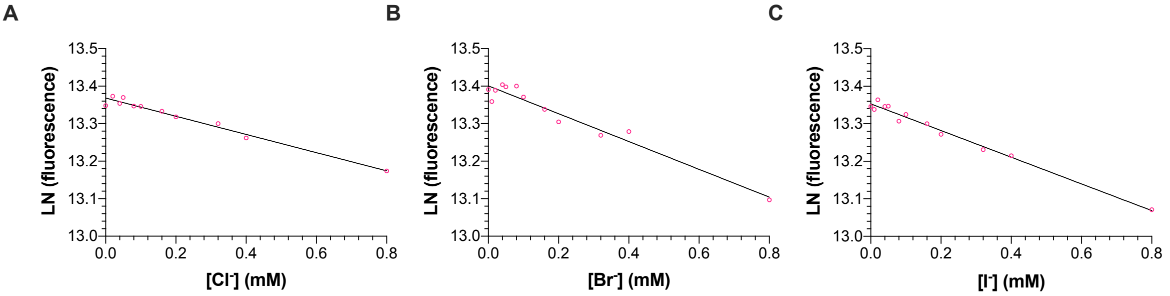

Figure S2: Standard curves for the lucigenin assay. Natural logarithms of fluorescence are plotted against concentrations of **A)** chloride, **B)** bromide, and **C)** iodide (n=1). Detection limits were determined as concentrations corresponding to fluorescence values that are triple the standard deviation of the intercept from the linear fit using Origin 6.1 (OriginLab, MA, USA). These values are 49 µM for chloride, 63 µM for bromide, and 35 µM for iodide. Detection limits are summarised in Table 1.

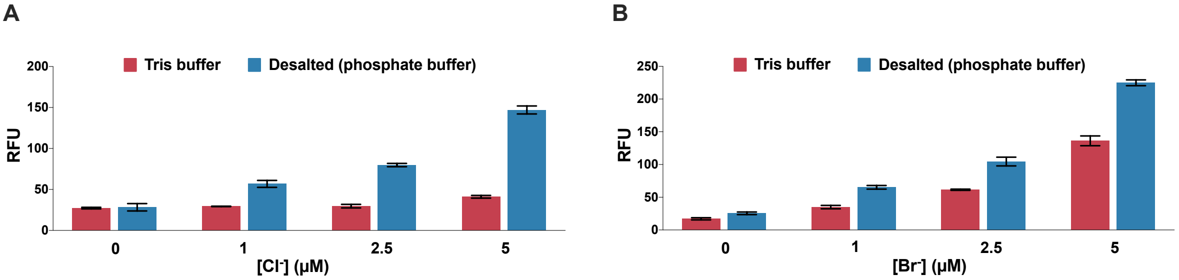

Figure S3: Inhibition of the HOX assay by Tris-H_2SO_4. The *Ci*VCPO originally contained 50 mM Tris-H_2SO_4 which inhibited the HOX assay for both **A)** chloride and **B)** bromide. The final concentration of Tris-H_2SO_4 in the assay mixture was 2.5 mM. Desalting into phosphate buffer (50 mM, pH 8.0) using PD10 columns significantly increased fluorescence for both chloride and bromide, but the effect was more pronounced for chloride. Values plotted are means with standard deviation (n=3). After we made this observation, the *Ci*VCPO was always dialysed against phosphate buffer before use, dramatically improving assay sensitivity. GraphPad Prism was used for plotting the data.

Section S1 Detailed Methods

Method S1.1 Expression and purification of *Ci*VCPO

The vanadium-dependent chloroperoxidase from *Curvularia inaequalis* (*Ci*VCPO) was expressed from the pBADVCPO vector as previously described.[2] The sequence of the recombinant *Ci*VCPO is given in Section S1.9. The vector was transformed into chemo-competent *E. coli* BL21(DE3) cells and colonies selected on LB agar containing 100 µg/ml ampicillin. For expression, 1 l of LB medium containing 100 µg/ml ampicillin was inoculated with 10 ml of an overnight culture grown from a single colony in the same medium. Cultures were incubated (180 rpm) at 37 °C until an optical density (600 nm) of 0.4-0.6 was reached. Expression was then induced by the addition of 0.02 % L-arabinose, followed by incubation at 25 °C for 24 h (160 rpm). Cells were harvested by centrifugation at 4500 *g* for 30 min (4 °C) and resuspended in 1 ml (per gram of cells) cold 50 mM Tris-H_2SO_4 (pH 8.1) containing 2 mg/ml lysozyme, 1 mg/ml DNaseI, and cOmplete™ protease inhibitor cocktail (Roche, Mannheim, Germany). Cells were then lysed on ice by ultrasonication (three cycles of 5 min at 60 % power and 50 % cycle setting) using a SONOPULS HD 2070 (BANDELIN electronic GmbH & Co. KG, Berlin, Germany). The suspension was allowed to cool on ice for five min between sonication cycles. The *E. coli* lysate was then clarified at 4 °C by centrifugation at 10,000 *g* for 1 h. The clarified lysate was transferred to a new tube, thoroughly mixed with an equal volume of 2-propanol and incubated at 60 °C for 20 min. Precipitated proteins were removed by centrifugation at 10,000 *g* for 30 min (4 °C). The *Ci*VCPO was then purified by chromatography on a 5 ml DEAE Sephacel column equilibrated with 50 mM Tris-H_2SO_4 (pH 8.1). The column was washed with 25 ml of 50 mM Tris-H_2SO_4 (pH 8.1) and then with 25 ml of the same buffer containing 100 mM NaCl. Protein was eluted from the column using 50 mM Tris-H_2SO_4 (pH 8.1) containing 1 M NaCl. The eluate (30 ml) was dialysed three times (twice for 4 h and then once overnight) against 4.5 l of 50 mM Tris-H_2SO_4 (pH 8.1) supplemented with 100 µM sodium orthovanadate, which converts the purified apoenzyme to the active vanadium-bound holoenzyme.[2-3] After finding that Tris-H_2SO_4 buffer inhibited the HOX assay (Figure S3), the purified *Ci*VCPO was always first dialysed against 50 mM sodium phosphate (pH 8.0) before use.

Method S1.2 Determining the specific activity of *Ci*VCPO

Specific activities of *Ci*VCPO preparations were determined using the monochlorodimedone assay. Bromination of monochlorodimedone results in a decrease in absorbance at 290 nm ($\Delta\varepsilon$ = 20,000 $M^{-1}cm^{-1}$).[4] Reactions (600 µl) containing 42 µM monochlorodimedone, 100 µM bromide, 8.8 mM H_2O_2, and 1 mM orthovanadate in 20 mM phosphate buffer (pH 6.0) were initiated by addition of 10 µl purified *Ci*VCPO. The decrease in absorbance at 290 nm was monitored using a 1 cm-pathlength quartz cuvette and a JASCO V-550 spectrophotometer (JASCO, MD, USA). Specific activity was expressed as µmol monochlorodimedone brominated/min/(mg *Ci*VCPO).

Method S1.3 Expression and purification of recombinant DhaA and DhlA

Recombinant His-tagged DhaA and DhlA were expressed from the vectors pET21b-DhaA and pET11a-DhlA as previously described.[5] Sequences of the recombinant proteins are given in Section S1.9. The plasmids were transformed into chemo-competent *E. coli* BL21(DE3) cells and colonies selected on LB agar plates containing 100 µg/ml ampicillin. For expression, 1 l of LB medium containing 100 µg/ml ampicillin was inoculated with 10 ml of an overnight culture grown from a single colony in the same medium. Cultures were incubated (180 rpm) at 37 °C until an optical density (600 nm) of 0.4-0.6 was reached. Expression was then induced by the addition of isopropyl β-D-1-thiogalactopyranoside (0.5 mM), followed by overnight incubation at 25 °C (160 rpm). Cells were harvested at 4 °C by centrifugation at 4,500 *g* for 30 min and resuspended in 1 ml (per gram of cells) cold 20 mM phosphate buffer (pH 7.5) containing 1 mg/ml lysozyme and 1 mg/ml DNaseI. Cells were lysed on ice by ultrasonication (three cycles of 5 min at 60 % power and 50 % cycle setting) using a SONOPULS HD 2070 (BANDELIN electronic GmbH & Co. KG, Berlin, Germany). The lysate was clarified at 4 °C by centrifugation at 10,000 *g* for 1 h. The recombinant His-tagged proteins were purified by immobilised metal-affinity chromatography using Roti®garose-His/Ni Beads and the manufacturer's protocol (Carl Roth GmbH + Co. KG, Karlsruhe, Germany). Purified proteins (~6 ml) were dialysed three times (twice for 4 h and then once overnight) against 4.5 l of 50 mM phosphate buffer (pH 8.0) to remove chloride and imidazole before being used in dehalogenase reactions.

Method S1.4 Fluorescence (HOX) assay using *Ci*VCPO and aminophenyl fluorescein

HOX assay reactions were 40 µl in volume and contained 2 mM H_2O_2, 25 µM aminophenyl fluorescein, 1 mM sodium orthovanadate, 2.5 U/ml *Ci*VCPO, and various halide concentrations in 20 mM sodium phosphate (pH 6.0 for chloride and bromide, pH 6.5 for iodide). We always performed at least three and up to nine replicates of assays. Reactions were incubated at room temperature for 60 min before fluorescence at 525 nm was measured by excitation at 488 nm using a Varioskan™ LUX plate reader (Thermo Fisher Scientific, Vantaa, Finland). Black 384-well plates were always used to prevent crosstalk between adjacent wells. Standard curves for chloride, bromide, and iodide were prepared by diluting stock solutions (2 µl) into a master mix containing all other assay components (38 µl). The standard curves plotted in Figure 1 show the final halide concentrations in the 40 µl reactions, not the concentrations of the stock solutions. The slightly higher background for iodide (Figure 1) results from the pH 6.5 buffer used. Sample volume could be increased from 2 µl to 30 µl by using a more concentrated master mix if desired.

Method S1.5 Iwasaki assay

The concentration of halide ions in 100 µl samples was analysed using the method developed by Iwasaki and co-workers.[6] Samples were reacted with mercuric thiocyanate and ferric ammonium sulfate and absorbance at 460 nm measured using a Sunrise microplate reader (Tecan Austria GmbH, Grödig, Austria). Calibration curves were prepared for chloride, bromide, and iodide. Detection limits were defined as the blank value plus triple the standard deviation from the blank (Figure S1).

Method S1.6 Lucigenin assay

An indicator stock solution (4 mM) was prepared by dissolving 2 mg of lucigenin in 1 ml of deionised water. Halides were then diluted to final concentrations between 0 mM and 10 mM in 20 mM sodium phosphate (pH 8.0, 1 ml) containing 3.92 µM lucigenin. The fluorescence of lucigenin solutions at 503 nm was determined using a FluoroMax-4P spectrofluorometer (HORIBA Scientific, NJ, USA) with a 150-W xenon arc lamp as 368 nm excitation light source. All measurements were carried out at 25 °C in square quartz cuvettes with a 1-cm pathlength. Natural logarithms of fluorescence were plotted against halide concentrations between 0 mM and 0.8 mM for chloride, bromide, and iodide (Figure S2). Detection limits were determined as concentrations corresponding to fluorescence values that are triple the standard deviation of the intercept from the linear fit using Origin 6.1 (OriginLab, MA, USA).

Method S1.7 Dehalogenase assays

To demonstrate a linear relationship between the amount of dehalogenase product formed and fluorescence obtained using the HOX assay, different concentrations of 1-bromobutane were completely hydrolysed using DhaA. Reactions (1 ml) containing 0.01 mg/ml DhaA and 1-bromobutane (0 to 2.5 mM) in 50 mM sodium phosphate (pH 8.0) were incubated at 30 °C, shaking at 800 rpm, for 1 h. After incubation, 10 µl phosphoric acid (42.5 %) was added to terminate the reactions.[7] Next, 10 µl aliquots of the terminated reactions were diluted 1/100 into 50 mM phosphate buffer (990 µl, pH 8.0). This dilution was performed in triplicate for each sample. Adding 4 µl of each diluted sample to a 36 µl HOX assay master mix resulted in an overall dilution of 1/1000 into the reaction mixture. Reactions were incubated at room temperature for 60 min before measuring and plotting fluorescence against the original concentrations of 1-bromobutane (Figure 2A).

A similar approach was followed for determining the specific activities of the recombinant dehalogenases DhaA and DhlA. First, a series of standards (1 ml) containing chloride (0 to 25 mM) or bromide (0 to 3 mM) in 50 mM sodium phosphate (pH 8.0) was prepared. Standards were treated in the same way as dehalogenase reactions by incubating at 30 °C for 1 h (800 rpm), adding 10 µl phosphoric acid (42.5 %), and diluting 10 µl aliquots 1/100 into 50 mM sodium phosphate (990 µl, pH 8.0). The HOX assay was then performed by adding 4 µl of the diluted standards to 36 µl of master mix. The initial standards were thus diluted 1/1000 into the final assay mixtures. After incubation at room temperature for 60 min, fluorescence was measured and plotted against the concentrations of the original standards. The linear calibration curves obtained (Figure 2B and C) were then used to quantify the amount of chloride or bromide released by DhaA and DhlA reactions.

All dehalogenase reactions for calculation of specific activities were performed in triplicate in 1 ml of 50 mM sodium phosphate (pH 8.0). Reactions contained 10 mM of either 1,2-dibromoethane, 1,2-dichloroethane, 1,3-dichloropropane, 1-bromo-3-chloropropane, or 1-bromohexane as substrate and were initiated by the addition of 10 µg DhaA or DhlA (final concentrations of 0.01 mg/ml). Reactions were incubated at 30 °C by shaking at 800 rpm for 30 to 90 min and then terminated, diluted, and assayed exactly as for the calibration standards. For each of three replicate dehalogenase reactions, three dilutions and HOX assays were performed, resulting in a total

of nine assay replicates for each combination of dehalogenase and substrate. The concentrations of halides produced were calculated using standard curves (Figure 2B and 2C) and used to express specific activities as nmol halide formed/s/(mg dehalogenase). The remaining volumes of the terminated dehalogenase reactions were used for calculation of specific activities using a GC-MS method.

Method S1.8 GC-MS analysis of dehalogenase reaction products

Each of three replicate dehalogenase reactions was analysed by GC-MS once, resulting in three replicates for each combination of dehalogenase and substrate. Due to the limited throughput of the GC-MS method, more replicates could not be analysed. Reaction products were quantified by comparison of product peak areas to standard curves for 2-bromoethanol, 2-chloroethanol, 3-chloro-1-propanol, and 1-bromohexanol.

Samples (200 µl) of the terminated dehalogenase reactions were taken and extracted by adding 200 µl *tert*-butyl methyl ether and vortexing at maximum speed for 1 min. The organic phase was then separated from the aqueous phase by centrifugation at 13,000 *g* for 5 min. Samples (1 µl) were analysed using a GCMS-QP2010 SE device (Shimadzu, Duisburg, Germany) with a ZB-5MSi column (30m × 0.25 mm, thickness 0.25 µm). Injector temperature was 220 °C and a flow rate of 1.08 ml/min was used. Column temperature was initially at 33 °C for 8 min, increased at 10 °C/min until 150 °C, held at 150°C for 1 min, and finally increased at 25 °C/min until 200 °C was reached. Mass spectrometer ion source temperature was 200 °C and interface temperature was 220 °C. Calibration curves were generated by preparing a standard series of each alcohol product in 1 ml of 50 mM sodium phosphate (pH 8.0). These standards were then treated with 10 µl phosphoric acid (42.5 %) before 200 µl portions were extracted using 200 µl *tert*-butyl methyl ether and analysed by GC-MS. The retention time was 5.56 min for 2-bromoethanol (Figure S4), 3.31 min for 2-chloroethanol (Figure S5), 9.5 min for 3-chloro-1-propanol (Figure S6), and 11.51 min for 1-hexanol (Figure S7). Standard curves (plots of peak area against product concentration) are shown in Figure S8.

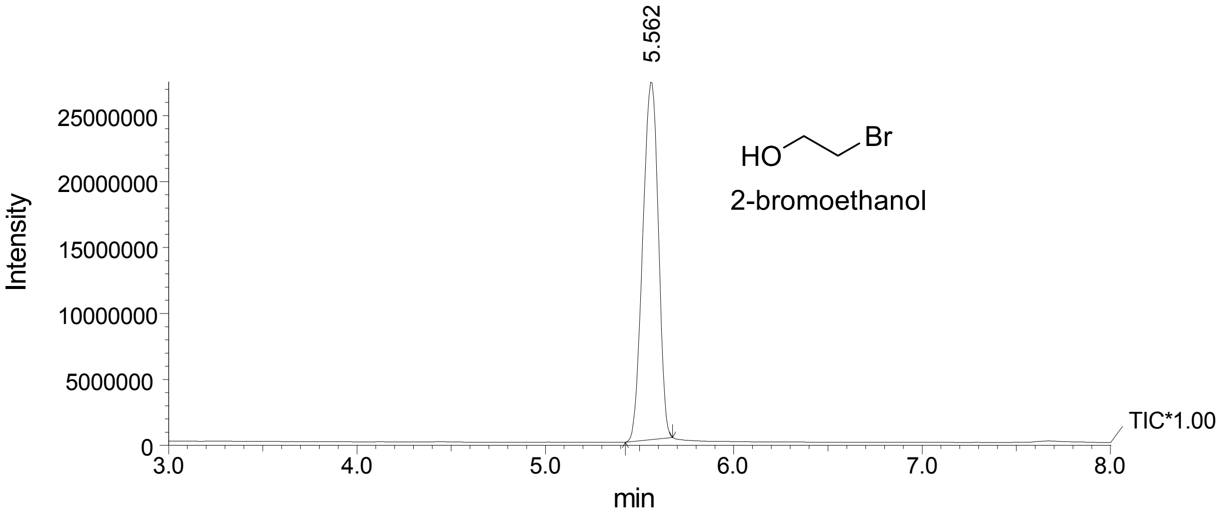

Figure S4: GC-MS analysis of a 2-bromoethanol product standard. The chromatogram shows the retention time of 5.56 min.

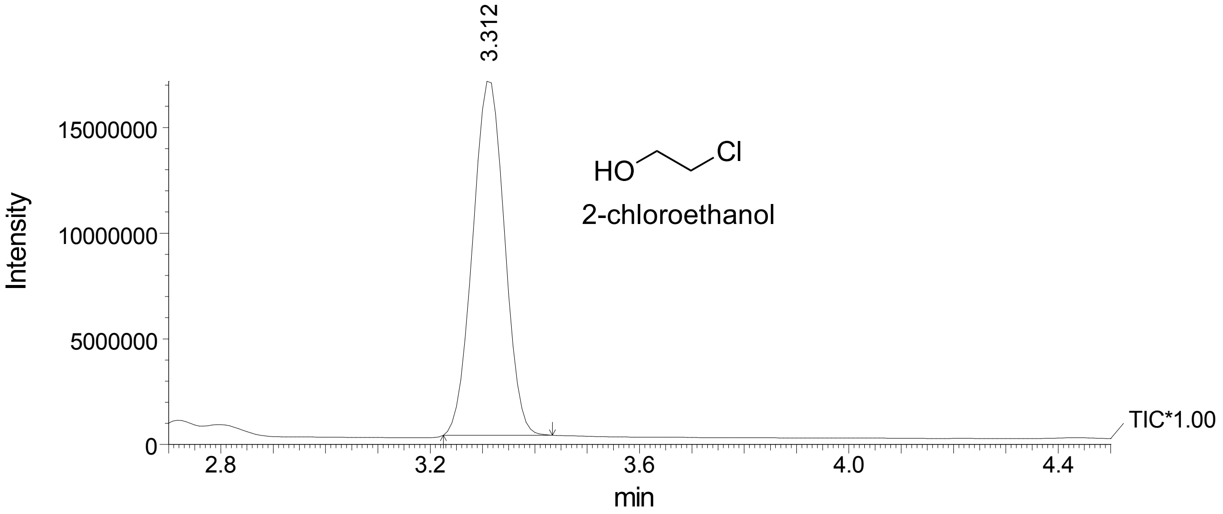

Figure S5: GC-MS analysis of a 2-chloroethanol product standard. The chromatogram shows the retention time of 3.31 min.

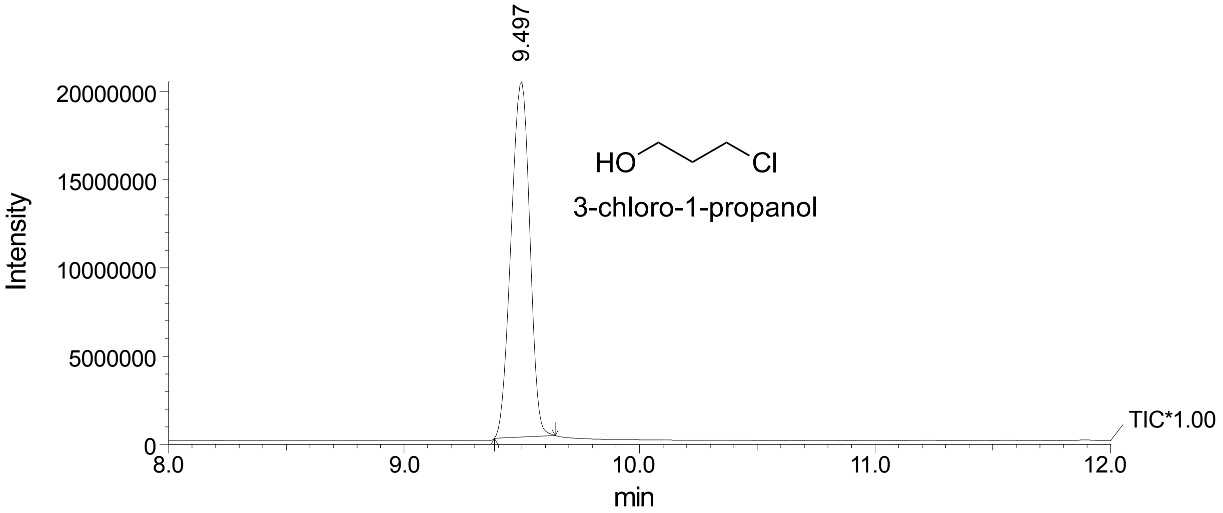

Figure S6: GC-MS analysis of a 3-chloro-1-propanol product standard. The chromatogram shows the retention time of 9.5 min.

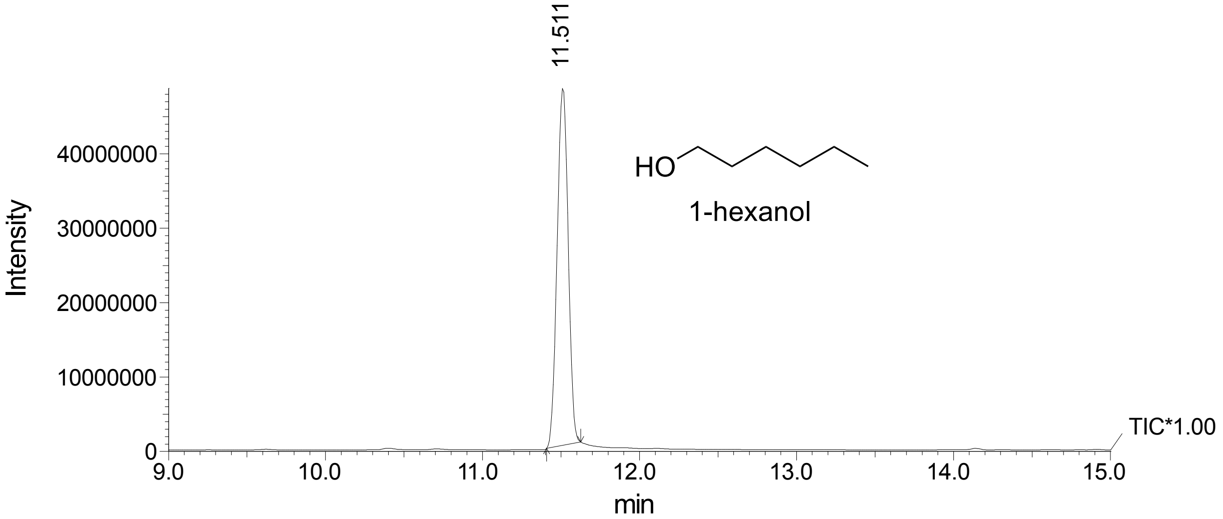

Figure S7: GC-MS analysis of a 1-hexanol product standard. The chromatogram shows the retention time of 11.51 min.

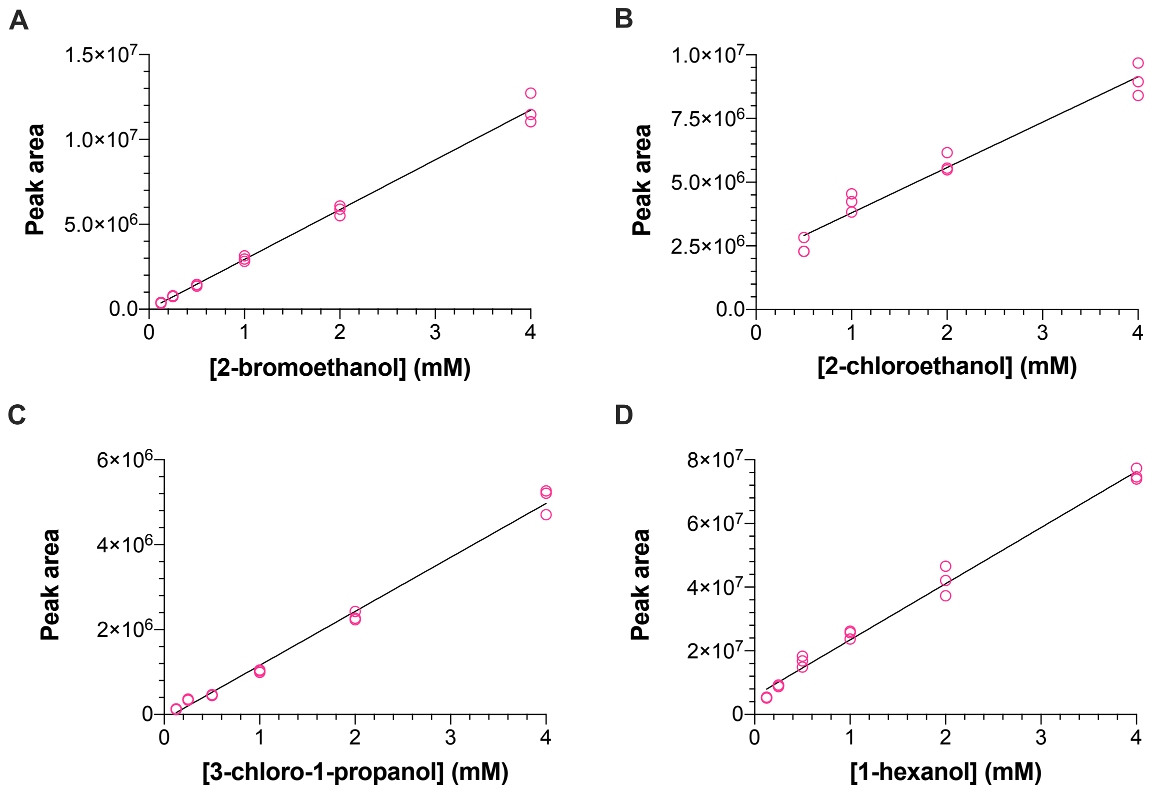

Figure S8: Standard curves for A) 2-bromoethanol, B) 2-chloroethanol, C) 3-chloro-1-propanol, and D) 1-hexanol. GC-MS peak areas are plotted against alcohol concentration. Each replicate is plotted as a separate data point (n=3). GraphPad Prism was used for plotting data and linear regression.

Section S1.9 Protein sequences

Vanadium-dependent chloroperoxidase from *Curvularia inaequalis*, expressed from pBADVCPO[2]

The gIII secretion sequence encoded by the pBAD vector is underlined. The sequence differs from PDB 1IDQ at two positions, highlighted in bold (D164A and P544R).[2]

>CiVCPO

MKKLLFAIPLVVPFYSHSTMASHMGSVTPIPLPKIDEPEEYNTNYILFWNHVGLELNRVTHTVGGPLTGPPLSAR
ALGMLHLAIHDAYFSICPPTDFTTFLSPDTENAAYRLPSPNGANDARQAVAGAALKMLSSLYMKPVEQPNPNPGA
NISDNAYAQLGLVLDRSVLEAPGGVDRESASFMFGE**A**VADVFFALLNDPRGASQEGYHPTPGRYKFDDEPTHPVV
LIPVDPNNPNGPKMPFRQYHAPFYGKTTKRFATQSEHFLADPPGLRSNADETAEYDDAVRVAIAMGGAQALNSTK
RSPWQTAQGLYWAYDGSNLIGTPPRFYNQIVRRIAVTYKKEEDLANSEVNNADFARLFALVDVACTDAGIFSWKE
KWEFEFWRPLSGVRDDGRPDHGDPFWLTLGAPATNTNDIPFKPPFPAYPSGHATFGGAVFQMVRRYYNGRVGTWK
DDEPDNIAIDMMISEELNGVNRDLRQPYDPTAPIEDQPGIVRTRIVRHFDSAWELMFENAISRIFLGVHWRFDAA
AARDILIPTTTKDVYAVDNNGATVFQNVEDIRYTTRGTRED**R**EGLFPIGGVPLGIEIADEIFNNGLKPTPPEIQP
MPQETPVQKPVGQQPVKGMWEEEQAPVVKEAP

DhlA from *Xanthobacter autotrophicus* GJ10, expressed from pET11a-DhlA[5b]

>DhlA

MINAIRTPDQRFSNLDQYPFSPNYLDDLPGYPGLRAHYLDEGNSDAEDVFLCLHGEPTWSYLYRKMIPVFAESGA
RVIAPDFFGFGKSDKPVDEEDYTFEFHRNFLLALIERLDLRNITLVVQDWGGFLGLTLPMADPSRFKRLIIMNAC
LMTDPVTQPAFSAFVTQPADGFTAWKYDLVTPSDLRLDQFMKRWAPTLTEAEASAYAAPFPDTSYQAGVRKFPKM
VAQRDQACIDISTEAISFWQNDWNGQTFMAIGMKDKLLGPDVMYPMKALINGCPEPLEIADAGHFVQEFGEQVAR
EALKHFAETEHHHHHH

DhaA from *Rhodococcus rhodochrous* NCIMB13064, expressed from pET21b-DhaA[5a]

>DhaA

MSEIGTGFPFDPHYVEVLGERMHYVDVGPRDGTPVLFLHGNPTSSYLWRNIIPHVAPSHRCIAPDLIGMGKSDKP
DLDYFFDDHVRYLDAFIEALGLEEVVLVIHDWGSALGFHWAKRNPERVKGIACMEFIRPIPTWDEWPEFARETFQ
AFRTADVGRELIIDQNAFIEGALPKCVVRPLTEVEMDHYREPFLKPVDREPLWRFPNELPIAGEPANIVALVEAY
MNWLHQSPVPKLLFWGTPGVLIPPAEAARLAESLPNCKTVDIGPGLHYLQEDNPDLIGSEIARWLPALHHHHHH

Section S2 Synthesis of aminophenyl fluorescein

Aminophenyl fluorescein was synthesised in two steps as described in the literature (Scheme S1).[8] Unless otherwise noted, chemicals of the highest purity available were purchased used without further purification. The purity of compounds **[2]** and **[3]** reported is > 95 %, according to NMR.

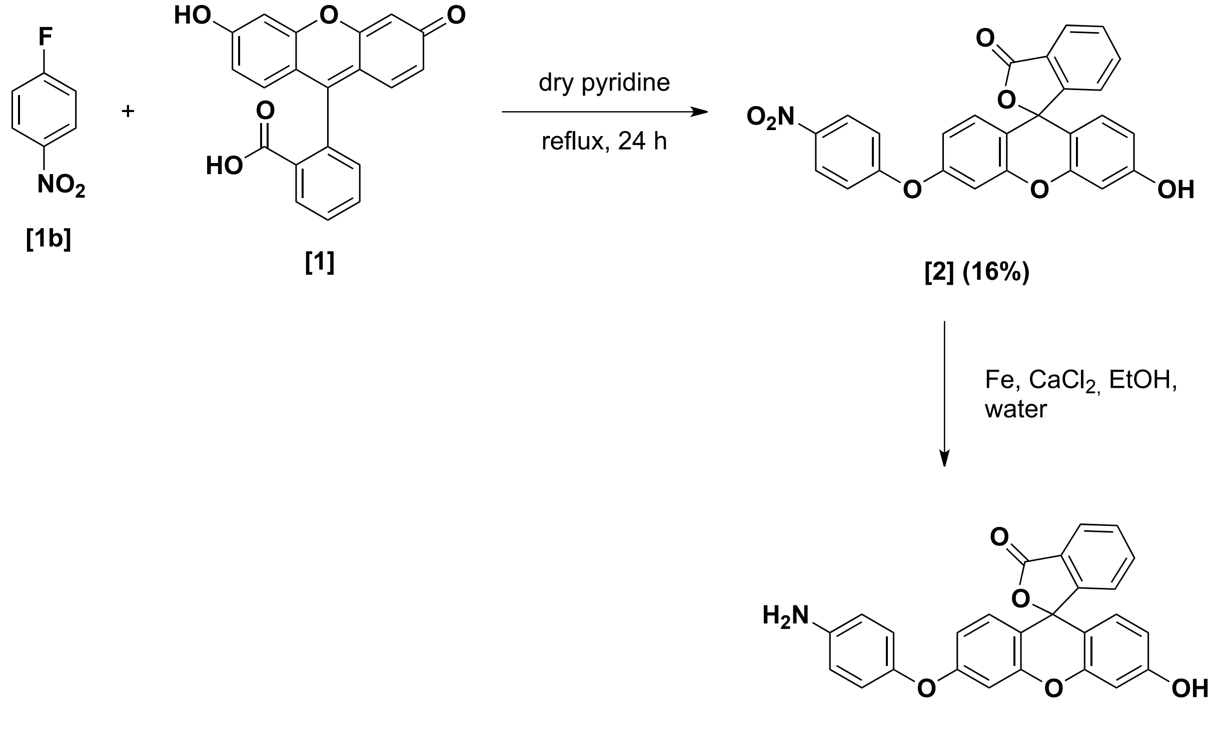

[3] (69%)

Scheme S1: Synthesis of aminophenyl fluorescein from fluorescein. Fluorescein **[1]** is refluxed with 4-fluoronitrobenzene **[1b]** in dry pyridine for 24 hours, yielding the nitrophenyl intermediate **[2]** which is purified by flash column chromatography and then reduced using iron powder to yield aminophenyl fluorescein **[3]**.

Method S2.1 NMR spectroscopy

NMR spectra were recorded on a Bruker Avance III HD 600 spectrometer equipped with a Prodigy BBO cryo probe (^{1}H: 600 MHz, ^{13}C: 151MHz; Bruker BioSpin GmbH, Rheinstetten, Germany). Chemical shifts are given in parts per million (ppm) and were calibrated with internal standards of the deuterium-labelled solvents $(CD_3)_2CO$ (^{1}H 2.05 ppm, ^{13}C 29.84 ppm) and CD_3CN (^{1}H 1.94 ppm, ^{13}C 1.32 ppm). NMR assignments of compounds were confirmed by ^{1}H - ^{1}H COSY, ^{1}H - ^{1}H, ^{1}H - ^{13}C, HSQC and ^{1}H - ^{13}C, HMBC, and by comparison to predicted spectra. Proton multiplicities are denoted by the following abbreviations: s (singlet), br s (broad singlet), d (doublet), br d (broad doublet), dd (doublet of a doublet), ddd (doublet of a doublet of a doublet), t (triplet), dt (doublet of a triplet), q (quartet), dq (doublet of a quartet), p (quintet), hep (septet), m (multiplet). Coupling constants (*J*) are presented in Hz (Hertz). Carbon multiplicities (suppressed CH coupling) are denoted by the following abbreviations: s (singlet), d (doublet), t (triplet) and q (quartet).

Method S2.2 Chromatographic methods

TLC was performed using silica gel 60 aluminium plates containing a fluorescent indicator from Merck and visualised under 254 nm UV light. Flash column chromatography (FC) was carried out on a Büchi Sepacore™ MPLC system (Büchi Labortechnik AG, Flawil, Switzerland) using silica gel 60 M (particle size 40-63 µm, 230-400 mesh ASTM; Macherey-Nagel, Düren, Germany).

Method S2.3 Melting point determination

Melting points were determined using a Büchi® Melting Point B-545 (Büchi Labortechnik AG, Flawil, Switzerland) and are uncorrected.

Method S2.4 Synthesis of 3'-hydroxy-6'-(4-nitrophenoxy)-3H-spiro[isobenzofuran-1,9'-xanthen]-3-one

Compound **[2]** was synthesized according to the following literature procedure.[8c] An oven-dried 500 ml three-neck flask equipped with a reflux condenser was flushed with argon three times using Schlenk-technique. Fluorescein **[1]** (free acid, 20 g, 60.18 mmol, 1.00 equiv.) and dry pyridine (200 ml) were added under an inert atmosphere and the resulting solution was stirred for 5 min. 4-Fluoronitrobenzene **[1b]** (33.97 g, 240.74 mmol, 4.00 equiv.) was further added and the reaction mixture was refluxed for 24 h under an argon atmosphere. After cooling to room temperature, the mixture was acidified with aqueous HCl and extracted with ethyl acetate (5 times, 200 ml). The organic extracts were combined, dried over $MgSO_4$, filtered and evaporated under reduced pressure to obtain the crude material as black oil. The pure product was obtained after purification by flash column chromatography (silica gel/crude material = 100/1, 20 %-50 % EtOAc in PE) as yellow crystals.

Yield: 16 % (4.50 g, 9.92 mmol)

Appearance: yellow crystals

Melting Point: 106-109 °C (decomposition); Lit[8b].: 104.5-106.5 °C (decomposition)

TLC: R_f (PE/EtOAc = 1/1) = 0.67

^{1}H NMR (600 MHz, acetone-*d*$_6$): δ = 6.67 (dd, *J* = 8.7, 2.3 Hz, 1H), 6.71 (d, *J* = 8.7 Hz, 1H), 6.78 (d, *J* = 2.3 Hz, 1H), 6.92 (dd, *J* = 8.7, 2.3 Hz, 1H), 6.95 (d, *J* = 8.7 Hz, 1H), 7.11 (d, *J* = 2.2 Hz, 1H), 7.28 (d, *J* = 9.3 Hz, 2H), 7.35 (d, *J* = 7.8 Hz, 1H), 7.84 (t, *J* = 7.5 Hz, 1H), 8.02 (d, *J* = 6.7 Hz, 1H), 8.30 (d, *J* = 8.8 Hz, 2H) ppm.

^{13}C-NMR (151 MHz, acetone-*d*$_6$): δ = 82.8, 103.4, 108.8, 111.2, 113.8, 116.7, 117.3, 119.4, 124.9, 125.6, 126.9, 127.6, 130.2, 131.0, 131.1, 136.3, 144.4, 153.0, 153.4, 153.7, 157.7, 160.6, 163.0, 169.3 ppm.

NMR spectra for compound **[2]** are shown in Figures S9-S12.

Comment: The ^{1}H-NMR is in accordance with the literature.[8b]

Method S2.5 Synthesis of 3'-(4-aminophenoxy)-6'-hydroxy-3H-spiro[isobenzofuran -1,9'-xanthen]- 3-one

The fluorophore APF **[3]** was synthesized according to the following literature procedure.[8a] A 50 ml round bottom flask was charged with the nitro compound **[2]** (4.50 g, 9.92 mmol, 1.00 equiv.) and EtOH (17.5 ml), water (8.5 ml), iron powder (1.66 g, 29.77 mmol, 3.00 equiv.) and $CaCl_2$ (1.10 g, 9.92 mmol, 1.00 equiv.) were added. The resulting black suspension was stirred at 60 °C until TLC analysis indicated full conversion of the starting material (2 h) and was then filtered over a short pad of celite. The solids were thoroughly washed with EtOAc and the solvent removed under reduced pressure. The pure product was obtained by flash column chromatography (dry load, silica gel/crude material = 100/1, 2 %-10 % MeOH in DCM) to obtain the product as beige solid.

Yield: 69 % (2.91 g, 6.87 mmol)

Appearance: beige solid

Melting Point: 152-155 °C (decomposition); Lit[8b].: 153-155 °C (decomposition)

TLC: R_f ($CHCl_3$/MeOH = 97/3) = 0.30

^{1}H NMR (600 MHz, acetonitrile-*d*$_3$): δ = 4.14 (br s, 2H), 6.55 (dd, *J* = 8.7, 2.5 Hz, 1H), 6.61 (dd, *J* = 8.8, 2.5 Hz, 1H), 6.64 (d, *J* = 8.7 Hz, 1H), 6.67 (s, 1H), 6.67 – 6.71 (m, 3H), 6.71 (d, *J* = 8.8 Hz, 1H), 6.85 (d, *J* = 8.8 Hz, 1H), 7.19 (d, *J* = 7.6 Hz, 1H), 7.68 (td, *J* = 7.5, 1.0 Hz, 1H), 7.74 (td, *J* = 7.5, 1.2 Hz, 1H), 7.97 (dt, *J* = 7.7, 1.0 Hz, 1H) ppm.

^{13}C-NMR (151 MHz, acetonitrile-*d*$_3$): δ = 83.7, 103.5, 104.6, 111.6, 113.4, 113.6, 113.8, 116.5, 122.5, 124.9, 125.7, 127.5, 130.3, 130.4, 131.0, 136.4, 146.5, 147.0, 153.2, 153.2, 153.9, 160.0, 162.4, 170.0 ppm.

NMR spectra for compound **[3]** are shown in Figures S13-S16.

Comment: The ^{1}H-NMR and melting point are in accordance with the literature.[8b, 8c]

Section 2.1 NMR spectra

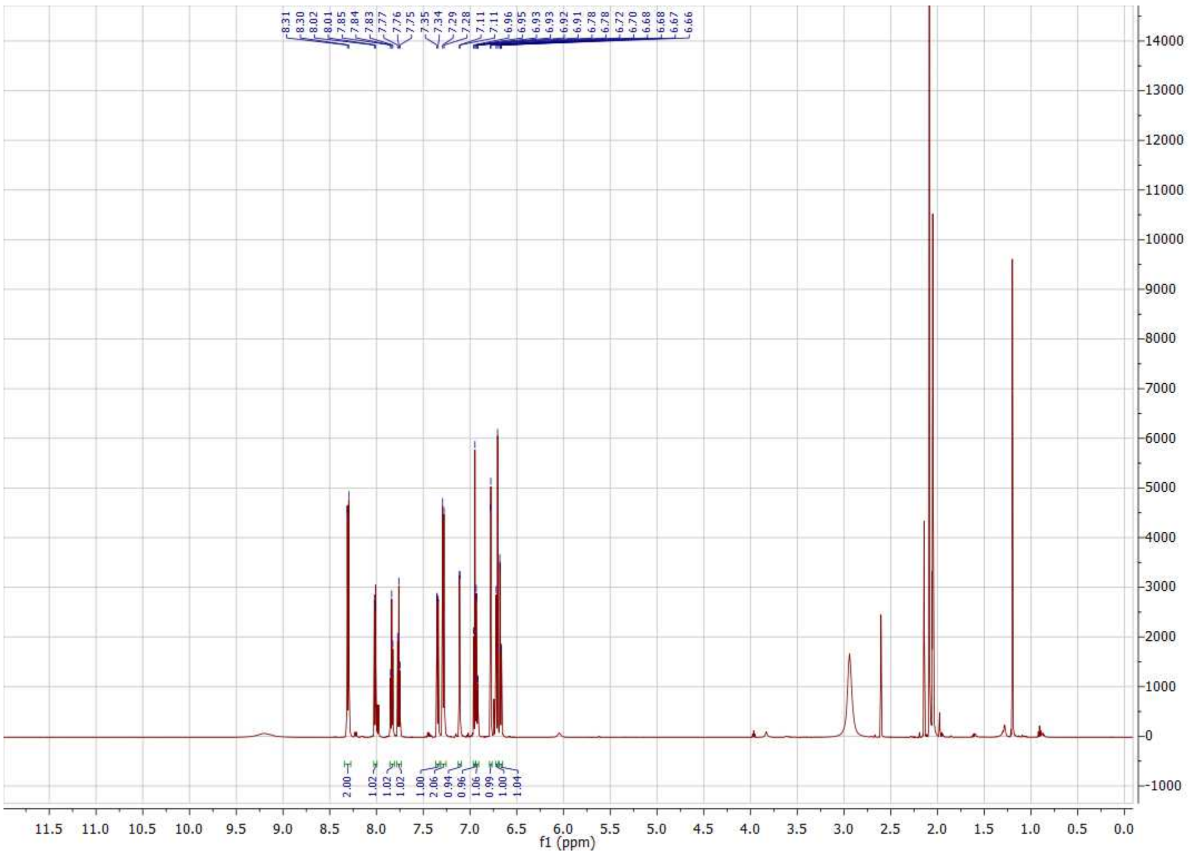

Figure S9: ^{1}H-NMR of compound **[2]**.

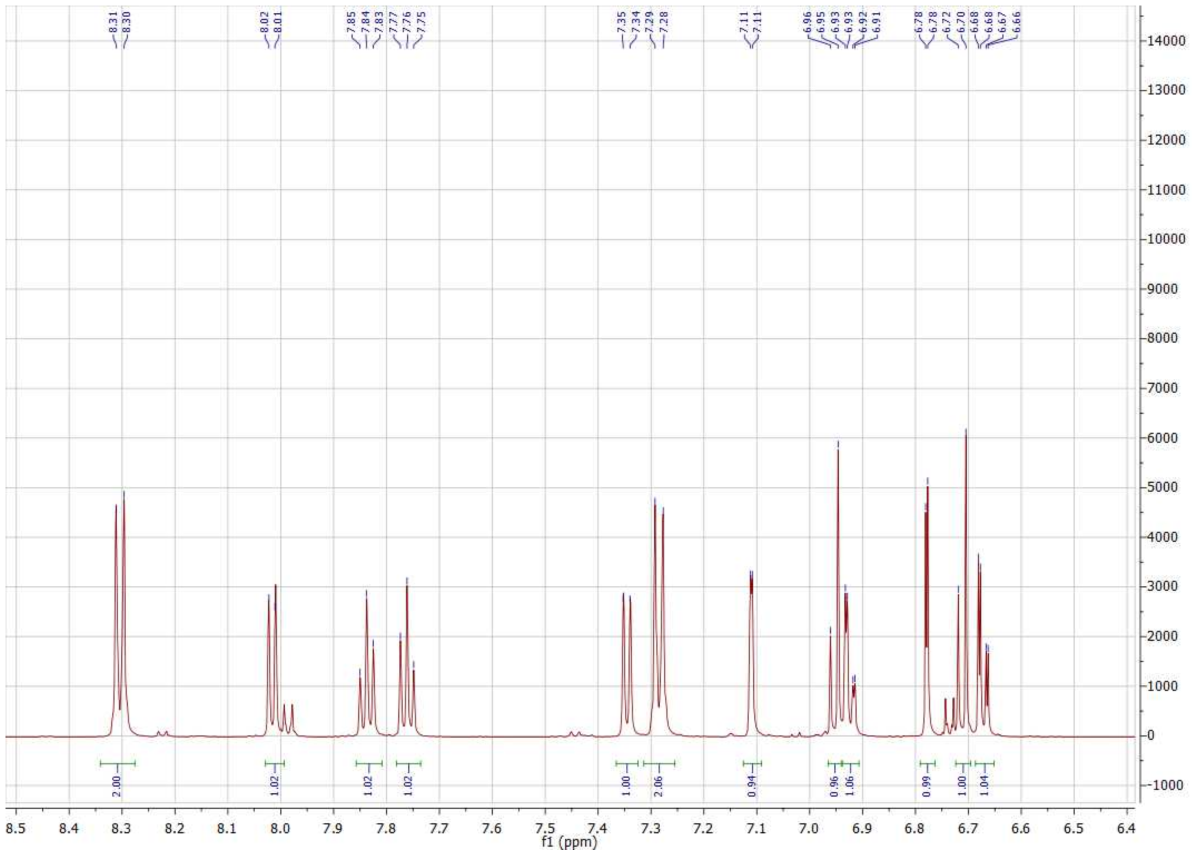

Figure S10: ^{1}H-NMR of compound **[2]** enlarged between 6.4 ppm and 8.5 ppm.

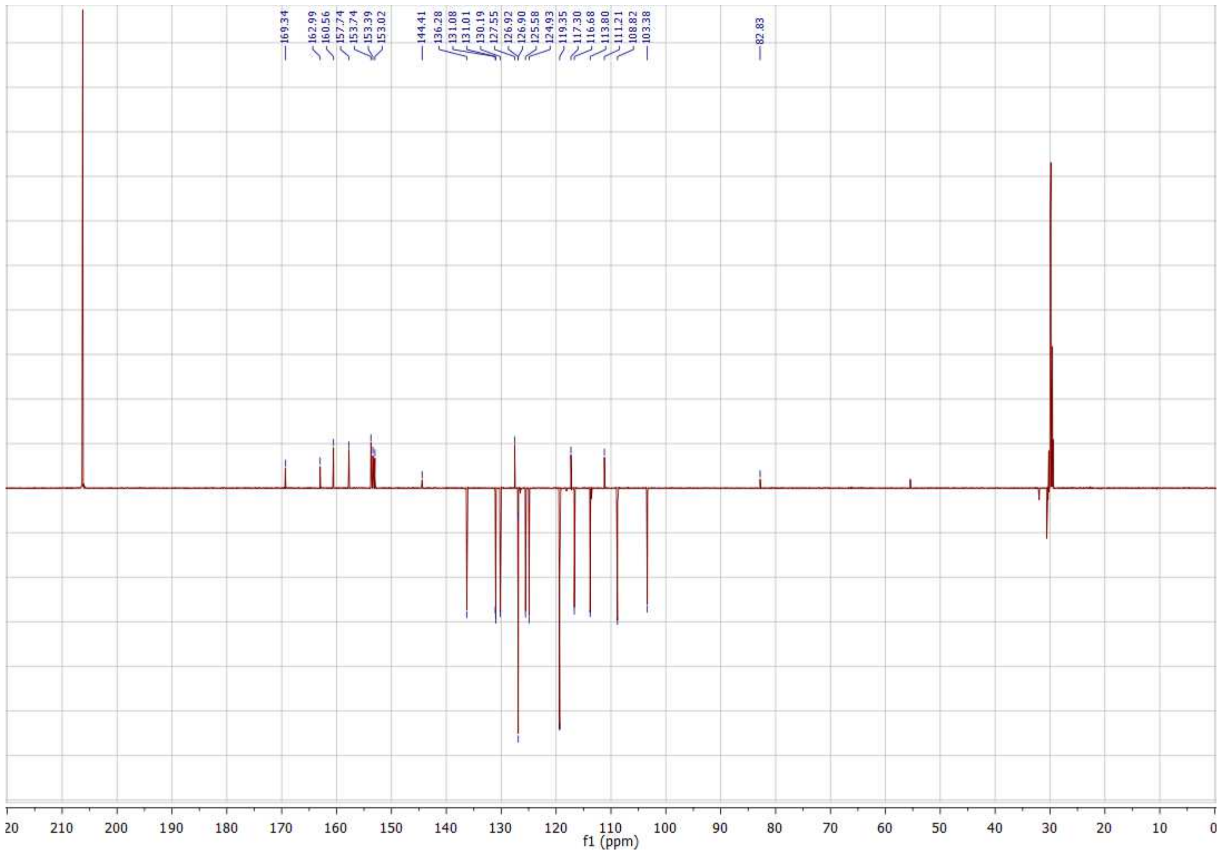

Figure S11: ^{13}C-NMR of compound **[2]**.

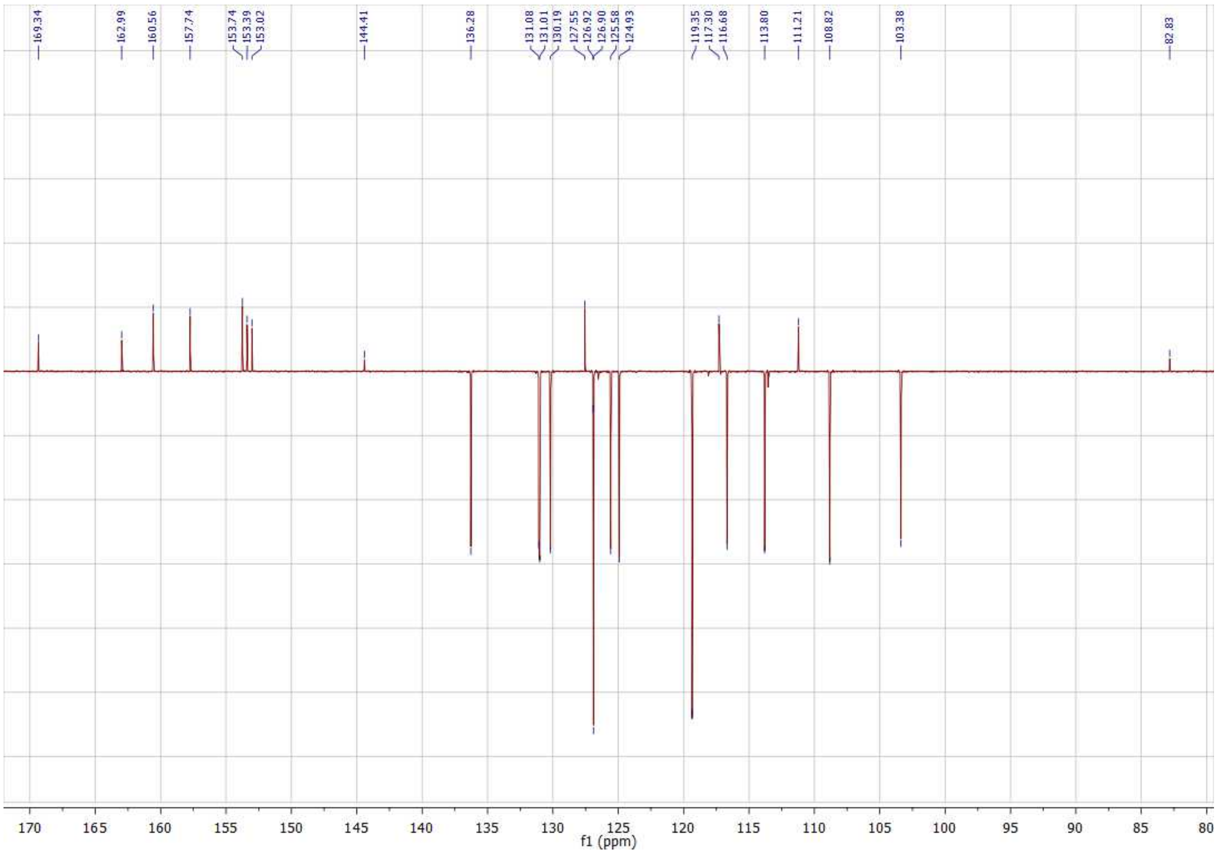

Figure S12: ^{13}C-NMR of compound **[2]** enlarged between 80 ppm and 175 ppm.

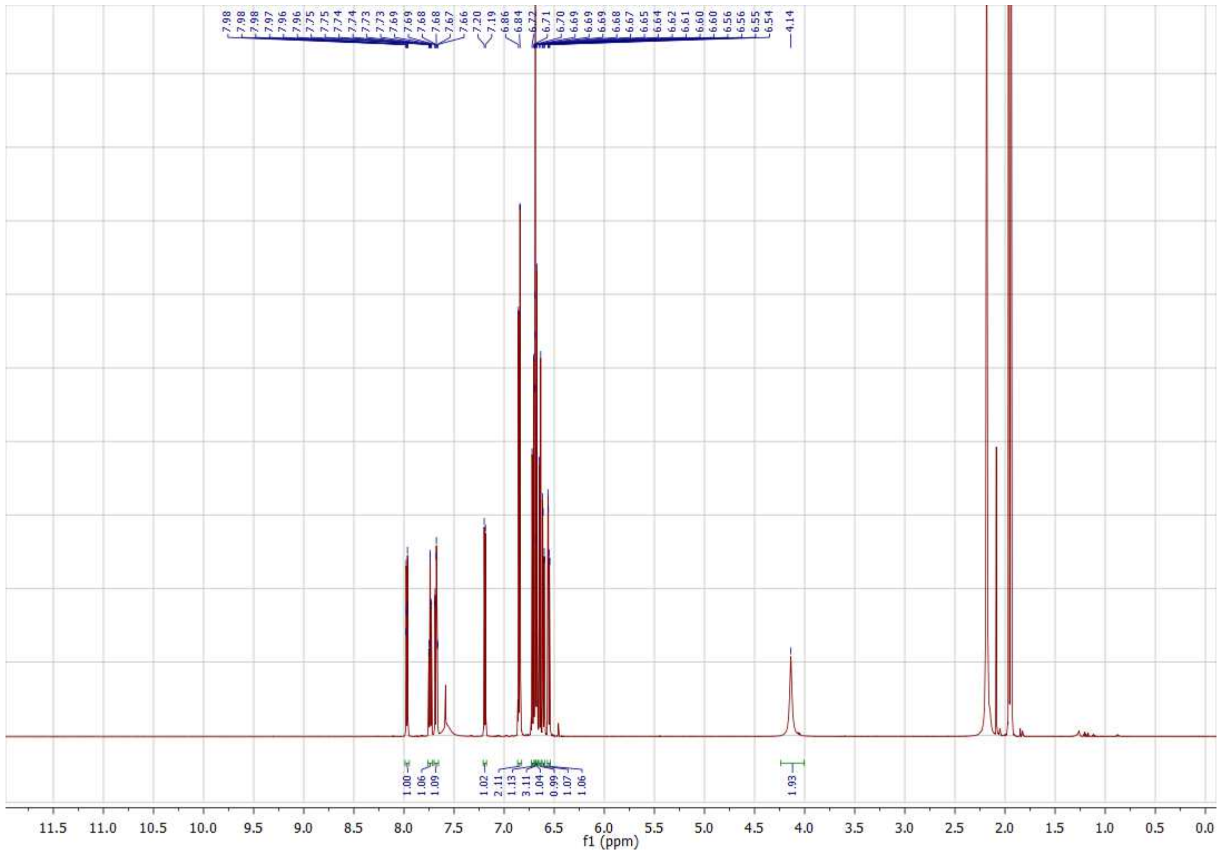

Figure S13: ^{1}H-NMR of compound **[3]**.

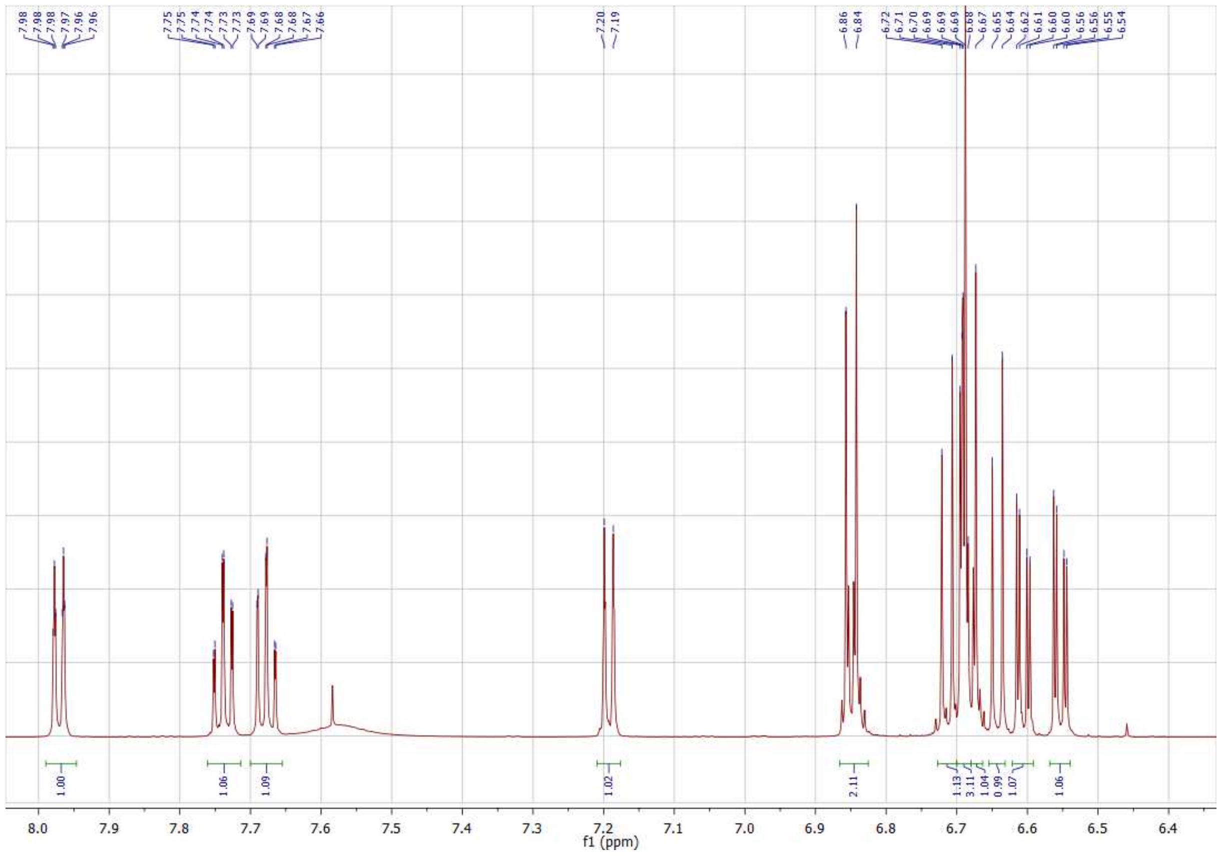

Figure S14: ^{1}H-NMR of compound **[3]** enlarged between 6.0 ppm and 8.5 ppm.

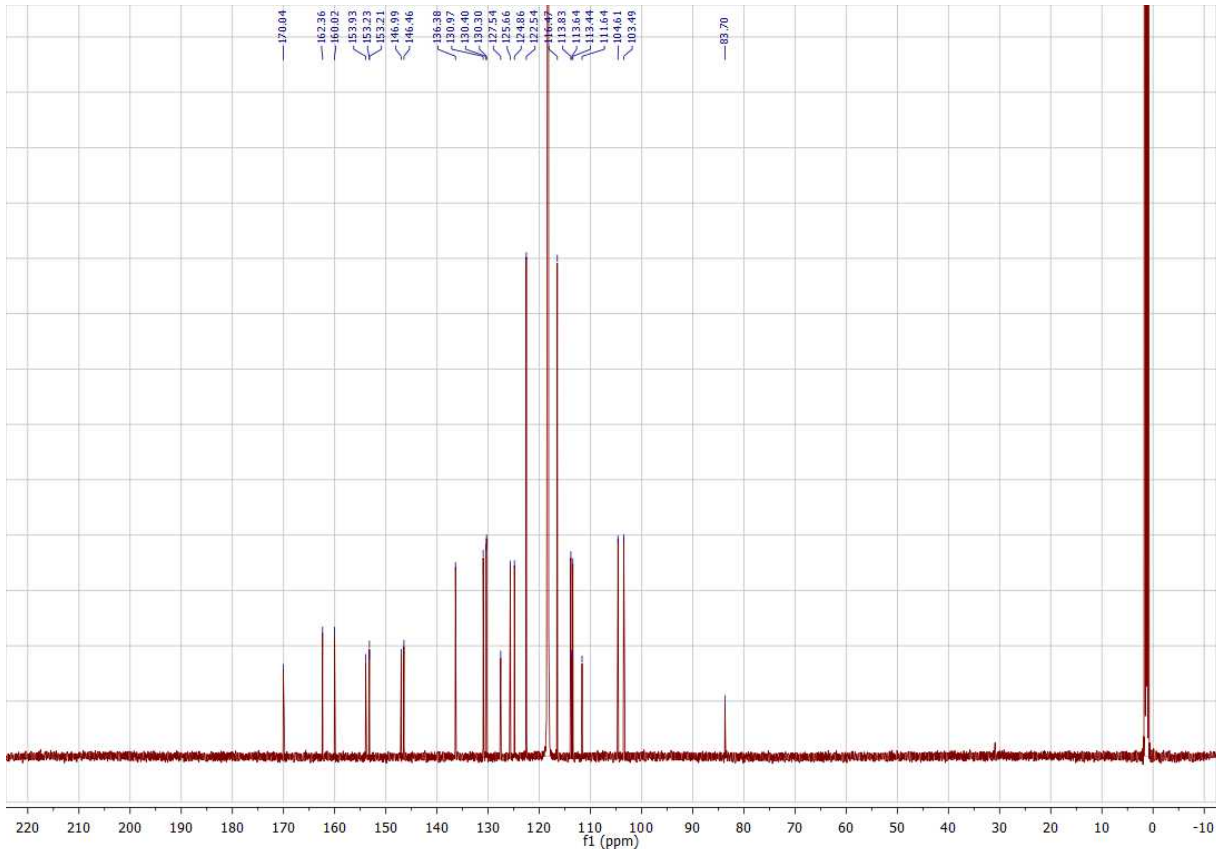

Figure S15: ^{13}C-NMR of compound **[3]**.

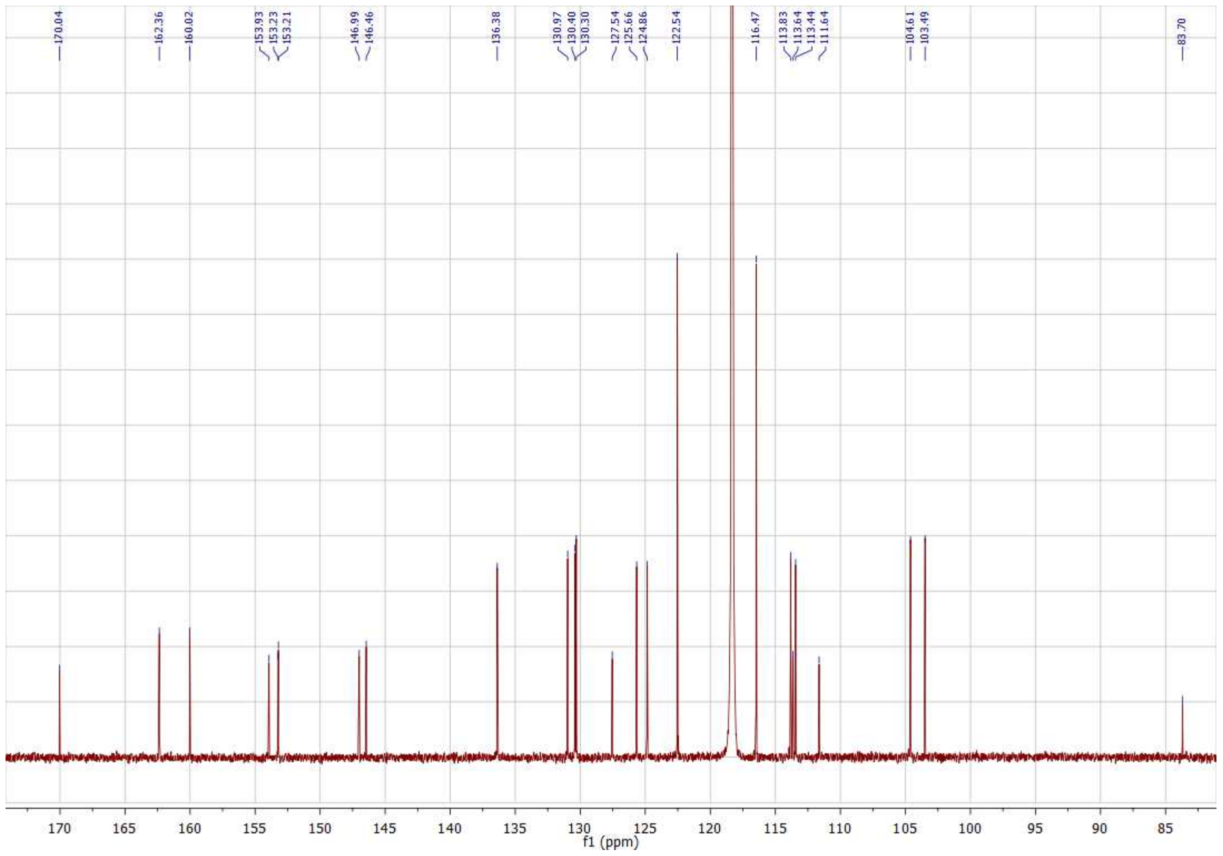

Figure S16: ^{13}C-NMR of compound **[3]** enlarged between 80 ppm and 180 ppm.

Section 2.2 List of abbreviations and chemical formulas

APF	aminophenyl fluorescein
Br^-	bromide
$CaCl_2$	calcium chloride
CD_3CN	deuterated acetonitrile
$(CD_3)_2CO$	deuterated acetone
$CHCl_3$	chloroform
*Ci*VCPO	vanadium-dependent chloroperoxidase from *Curvularia inaequalis*
Cl^-	chloride
COSY	correlated spectroscopy
DCM	dichloromethane
DhaA	dehalogenase from *Rhodococcus rhodochrous* NCIMB13064
DhlA	dehalogenase from *Xanthobacter autotrophicus* GJ10
EtOAc	ethyl acetate
EtOH	ethanol
equiv.	equivalents
HMBC	heteronuclear multiple-bond correlation spectroscopy
HOBr	hypobromous acid
HOCl	hypochlorous acid
HOI	hypoiodous acid
HOX	hypohalous acid
HSQC	heteronuclear single-quantum correlation spectroscopy
H_2O_2	hydrogen peroxide
I^-	iodide
MeOH	methanol
$MgSO_4$	magnesium sulfate
NMR	nuclear magnetic resonance spectroscopy
PE	petroleum ether
ppm	parts per million
rpm	revolutions per minute
TLC	thin layer chromatography
X^-	halide

References

[1] L. Jeske, S. Placzek, I. Schomburg, A. Chang, D. Schomburg, *Nucleic Acids Res.* **2019**, *47*, D542-D549.

[2] E. Fernández-Fueyo, S. H. H. Younes, S. van Rootselaar, R. W. M. Aben, R. Renirie, R. Wever, D. Holtmann, F. P. J. T. Rutjes, F. Hollmann, *ACS Catal.* **2016**, *6*, 5904-5907.

[3] Z. Hasan, R. Renirie, R. Kerkman, H. J. Ruijssenaars, A. F. Hartog, R. Wever, *J. Biol. Chem.* **2006**, *281*, 9738-9744.

[4] a) A. Butler, J. V. Walker, *Chem. Rev.* **1993**, *93*, 1937-1944; b) J. W. van Schijndel, E. G. Vollenbroek, R. Wever, *Biochim. Biophys. Acta* **1993**, *1161*, 249-256.

[5] a) P. Dvorak, S. Bidmanova, J. Damborsky, Z. Prokop, *Environ. Sci. Technol.* **2014**, *48*, 6859-6866; b) M. P. Fibinger, T. Davids, D. Böttcher, U. T. Bornscheuer, *Appl. Microbiol. Biotechnol.* **2015**, *99*, 8955-8962.

[6] I. Iwasaki, S. Utsumi, T. Ozawa, *Bull. Chem. Soc. Jpn.* **1952**, *25*, 226-226.

[7] J. Zhang, X. Cao, Y. Xin, S. Xue, W. Zhang, *World J. Microbiol. Biotechnol.* **2013**, *29*, 1791-1799.

[8] a) S. Chandrappa, K. Vinaya, T. Ramakrishnappa, K. S. Rangappa, *Synlett* **2010**, 3019-3022; b) K. Setsukinai, Y. Urano, K. Kakinuma, H. J. Majima, T. Nagano, *J. Biol. Chem.* **2003**, *278*, 3170-3175; c) G. Zhou, Y. Guan, *Chromatographia* **2016**, *79*, 319-325.
